# The influence of realistic 3D mantle viscosity on Antarctica’s contribution to future global sea levels

**DOI:** 10.1126/sciadv.adn1470

**Published:** 2024-08-02

**Authors:** Natalya Gomez, Maryam Yousefi, David Pollard, Robert M. DeConto, Shaina Sadai, Andrew Lloyd, Andrew Nyblade, Douglas A. Wiens, Richard C. Aster, Terry Wilson

**Affiliations:** ^1^ Earth and Planetary Sciences Department, McGill University, Montreal, Canada.; ^2^ Department of Geosciences, Pennsylvania State University, State College, PA, USA.; ^3^ Department of Earth, Geographic and Climate Sciences, University of Massachusetts Amherst, Amherst, MA, USA.; ^4^ Union of Concerned Scientists, Cambridge, MA, USA.; ^5^ Lamont Doherty Earth Observatory, Columbia University, New York, NY, USA.; ^6^ Department of Earth, Environmental, & Planetary Sciences, Washington University, St. Louis, MO, USA.; ^7^ Department of Geosciences, Warner College of Natural Resources, Colorado State University, Fort Collins, CO, USA.; ^8^ School of Earth Sciences, Ohio State University, Columbus, OH, USA.

## Abstract

The response of the Antarctic Ice Sheet (AIS) to climate change is the largest uncertainty in projecting future sea level. The impact of three-dimensional (3D) Earth structure on the AIS and future global sea levels is assessed here by coupling a global glacial isostatic adjustment model incorporating 3D Earth structure to a dynamic ice-sheet model. We show that including 3D viscous effects produces rapid uplift in marine sectors and reduces projected ice loss for low greenhouse gas emission scenarios, lowering Antarctica’s contribution to global sea level in the coming centuries by up to ~40%. Under high-emission scenarios, ice retreat outpaces uplift, and sea-level rise is amplified by water expulsion from Antarctic marine areas.

## Teaser

Solid Earth uplift reduces Antarctica’s contribution to sea-level rise under low warming but amplifies it under high warming.

## INTRODUCTION

With nearly 700 million people living in coastal areas and estimates of the cost of sea-level rise reaching ~14 trillion dollars by the end of the century, understanding the future contribution of the polar ice sheets to global sea levels in coming years is of critical societal importance (*1*, *2*). Ice-sheet changes take place on a range of spatiotemporal scales and depend on interactions with the surrounding atmosphere, ocean, and solid Earth (*3*). Large sectors of the Antarctic Ice Sheet (AIS) feature glaciers terminating in the ocean with their base lying below the local sea surface height (*4*). These marine-based sectors have long been considered susceptible to rapid, runaway retreat (*5*–*10*). Satellite and in situ observations indicate that marine sectors of the AIS are currently changing with possible runaway retreat already occurring (*11*). Modern instrumental records do not resolve the types of major AIS changes we may expect in the future, but geological evidence suggests that substantial marine ice-sheet retreat has caused multi-meter global sea-level rise at times in Earth history when the climate was warmer than present (*3*, *12*, *13*). Projections of future ice loss in these regions differ substantially among studies, however, and dominate the uncertainty in future sea-level projections (*1*, *14*–*17*).

**Fig. 1.**
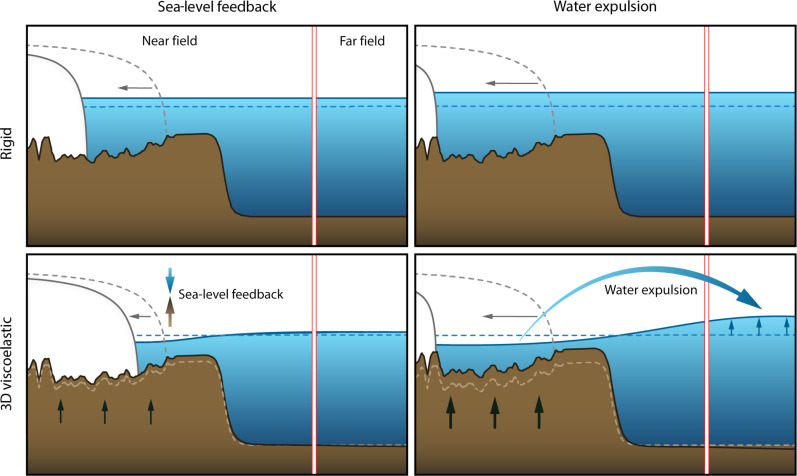
Schematic illustrating the sea-level feedback and water expulsion mechanisms. (Left column) Illustration of the sea-level feedback mechanism that dominates under low climate forcing whereby uplifting bedrock and lowering sea surface beneath marine sectors underlain by low viscosity reduce ice loss across the grounding line, leading to reduced far-field sea-level rise (bottom panel) compared to a scenario where these effects are excluded (top panel). (Right column) Illustration of the water expulsion mechanism whereby exposed ocean areas in Antarctica continue to rebound (bottom panel), expelling water out of Antarctica and increasing far-field sea level more relative to the scenario with a rigid bed (top panel). Note that both mechanisms are active in general, but under low climate forcing, the sea-level feedback dominates and GIA reduces WAIS contribution to GMSL rise. On the other hand, under strong climate forcing, the ice-sheet retreat is too fast to be strongly sensitive to the uplifting bedrock and the sea-level feedback is weaker so the ice sheet retreats in a similar way regardless of whether a rigid or 3D viscoelastically deforming Earth model is adopted, but substantial ice loss and bedrock uplift occurs in exposed marine areas with the 3D deforming Earth model. Hence, water expulsion is the dominant effect, enhancing Antarctica’s contribution to global mean sea-level rise. Figure by E. Goblet.

**Fig. 2.**
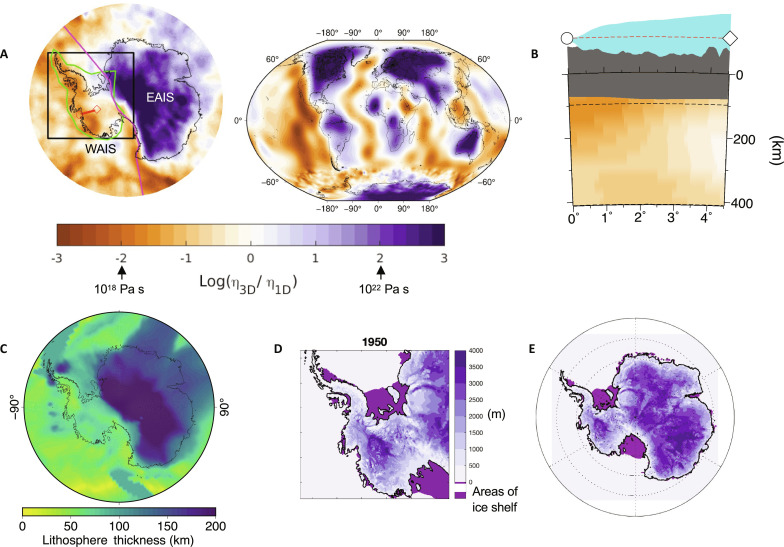
3D Earth structure beneath the AIS. (**A** and **B**) The constructed 3D Earth viscosity structure based on seismic tomography (*31*) in Antarctica and (*40*) globally, plotted (A) at 120-km depth and (B) along a vertical cross section beneath the Thwaites Glacier (shown by red lines in frame (A), and discussed in Figs. 3 and 4), using the same viscosity color scale. The vertical axis above the solid black line in (B) has been amplified to show variations in ice thickness and bedrock elevation. The gray area below the solid black line represents the lithospheric thickness along the cross section in the 3D model, while the dashed black line represents the 96-km lithospheric thickness of the 1D reference model. Viscosity variations are relative to a reference 1D radially varying viscoelastic model representing an Antarctica average with a viscosity of 10^20^ Pa s in the upper mantle from the base of the lithosphere down to 670-km depth. The black box in (A) shows the higher-resolution nested ice-sheet model simulation region (see Materials and Methods), while the green line outlines the area of grid refinement in the GIA model. The pink line defines the WAIS-EAIS division adopted in sea-level contribution calculations presented in Fig. 3 and figs. S1 and S2. (**C**) Lithospheric thickness in the 3D Earth model in kilometers. The initial grounded ice thickness in meters and location of floating ice shelves at year 1950 in the ice-sheet model are shown over the ice-sheet model’s (**D**) nested and (**E**) continental domains. Black lines in (D) and (E) indicate grounding line location.

Past and long-term future marine ice-sheet evolution in Antarctica depends on the evolving elevation of the solid Earth beneath the ice and on sea levels at the grounding line (*18*–*23*). Coupled ice sheet–glacial isostatic adjustment (GIA) models (also termed coupled ice sheet–sea level models in the literature) show that the future evolution of the ice sheet is sensitive to the underlying Earth structure, with models characterized by lower viscosity mantle and thinner lithosphere producing more and earlier uplift and relative sea-level fall beneath ice-sheet grounding lines across Antarctica (*20*, *21*, *23*). These outcomes are more effective at slowing or stopping future ice-sheet retreat on millennial timescales because ice flux is strongly sensitive to the thickness of ice at the grounding line, which is in turn proportional to water depth, and a fall in sea level therefore reduces the ice discharge across the grounding line—an effect termed “the sea-level feedback” (Fig. 1) (*18*). Model results have also highlighted the importance of including low viscosity beneath individual glaciers on centennial timescales (*24*, *25*). These models adopted a spherically symmetric Earth structure that is unlikely to represent the strength of ice–Earth–sea level feedbacks across the Antarctic continent, or even across West Antarctica. Global Navigation Satellite System (GNSS) inferences and seismic imaging indicate large lateral [three-dimensional (3D)] changes in Earth structure across the Antarctic continent (*26*–*32*), including estimated mantle viscosities as low at 10^18^ Pa s and a thinner lithosphere in parts of West Antarctica leading to viscous deformation on decadal timescales, rather than the millennial scales commonly adopted in Antarctic-wide modeling studies [e.g., (*33*, *34*)]. In contrast, seismic tomography models and tectonic reconstructions (*31*, *35*–*37*) suggest that much of the East Antarctic Ice Sheet (EAIS) sits atop a craton with cooler, more viscous mantle and thicker than average lithosphere (Fig. 2).

A recent study with a sea-level model with deformation of a purely elastic Earth coupled to a dynamic ice-sheet model showed the importance of elastic effects for the strength of the sea-level feedback on ice-sheet evolution (*22*). Not included in the study, however, is viscous deformation associated with modern and future ice loss, which has been shown to contribute up to 60% of the total predicted GIA by the end of the 21st century in low-viscosity regions such as the Amundsen Sea Embayment (*38*). Literature applying coupled ice sheet–GIA models with radially varying Earth structure and simplified Earth deformation treatments suggests that viscous deformation has the potential to affect future grounding line dynamics in West Antarctica (*20*, *21*, *23*–*25*, *39*). However, Earth structure varies markedly in Antarctica across small spatial scales, and up to now, no studies have robustly assessed the impact of 3D Earth structure on the AIS and global, spatially variable sea level incorporating both the elastic and viscous responses.

Here, we bring together the latest advances in coupled ice sheet–GIA modeling and solid Earth geophysics, informed by geophysical and geological observables, to assess the influence of GIA and 3D Earth structure on AIS evolution and its contribution to global sea-level changes in the coming centuries. To capture lateral variations in Earth structure, we combine the most recent seismic and geodetic records in Antarctica (*26*, *28*, *29*, *31*) with a global seismic tomography model (*40*) to build a global 3D model of Earth’s mantle viscosity and lithospheric thickness with 3-km spatial resolution beneath key areas of ice loss in Antarctica (Fig. 2; see Materials and Methods). This 3D viscoelastic Earth model serves as input to a 3D GIA model (*41*, *42*) that includes gravitational effects and rotation and deformation of an elastically compressible, viscoelastically deforming Earth (see Materials and Methods). The model includes migrating shorelines and state-of-the-art treatment of water expulsion (Fig. 1) in uplifting marine sectors of Antarctica that includes its impact on calculations of Antarctica’s contribution to global sea levels (*43*, *44*). To capture the sea-level feedback on grounding line migration (Fig. 1), we couple this 3D GIA model to the dynamic PSUICE3D ice sheet–shelf model (*15*) with a new coupling algorithm based on (*42*) but with the addition of nesting in the ice model to reach 5-km resolution across West Antarctica, matched with grid refinement in the GIA model to 3 km (resulting in a global computational grid with ~28 million grid nodes) to accurately represent the GIA response (Fig. 2) (*38*). The ice-sheet model setup (*15*) is informed by modern satellite constraints on mass balance as well as paleo–sea-level records from past warm periods, and adopts improved climate forcing from regional climate modeling (see Materials and Methods) with more finely resolved meteorology relative to previous coupled ice sheet–global GIA simulations. Results exploring resolution and Earth structure dependence of GIA predictions (*38*) are used to inform our GIA model setup (see Materials and Methods). Our coupled modeling framework represents a substantive advance in computational state-of-the-art, capturing future ice-Earth-global sea-level interactions at unprecedented detail and complexity.

We perform a suite of simulations considering a range of Earth rheologies (rigid, elastic, 1D viscoelastic, and 3D viscoelastic; see Materials and Methods), climate forcings (Representative Concentration Pathway scenarios RCP2.6, RCP4.5, and RCP8.5), and ice physics assumptions. We repeat all scenarios with consideration of ice shelf hydrofracture and the marine ice cliff instability mechanism [henceforth MICI; (*15*)] and considering only marine ice-sheet instability without MICI (henceforth MISI). By combining state-of-the-art treatment of sea level and ice physics, our findings quantify the importance of Earth rheology and structure for future projections of grounding line dynamics, solid Earth deformation, ice mass changes, sea levels, and coastlines around the globe.

## RESULTS

### Projections of Antarctica’s future contribution to global sea-level changes

**Fig. 3.**
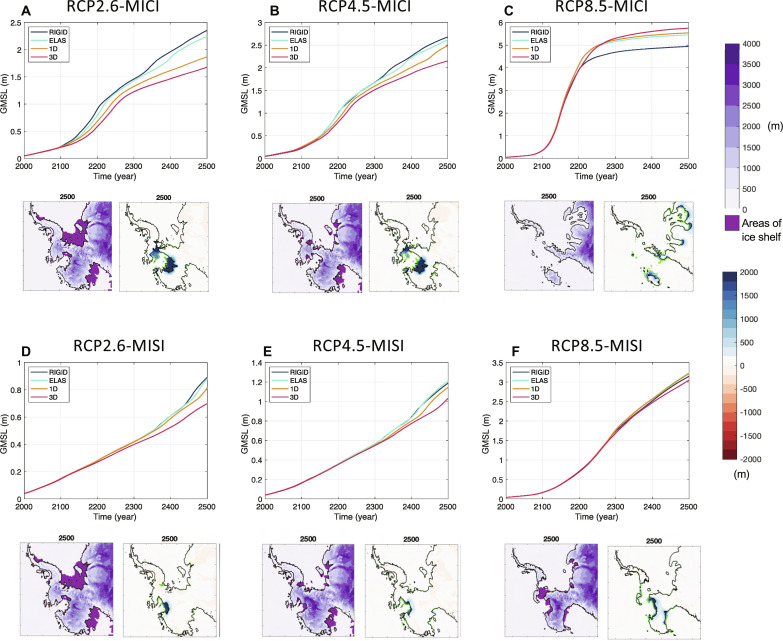
West Antarctic ice loss and contribution to global GMSL for a range of adopted Earth structure models, ice physics, and climate warming scenarios. Values given in the time series [plots in (**A** to **F**)] are from nested ice-sheet model simulations, just for the region of West Antarctica bounded by the pink line in Fig. 2A. Projected global mean sea level change (ΔGMSL) associated with WAIS evolution for six different ice physics and climate warming scenarios: RCP2.6 (A and D), RCP4.5 (B and E), and RCP8.5 (C and F), including MICI (A to C) and excluding MICI and considering MISI only (D to F). Colored lines represent simulations adopting four different assumptions of the Earth structure as indicated in the legend. Details of each of these models are described in Materials and Methods. ΔGMSL calculations shown here are described in Materials and Methods, and ΔGMSL calculations with a basic VAF approach are plotted in fig. S1. Beneath each time series plot in (A) to (F) are ice thickness for the 3D Earth model simulations (left, with corresponding color bar on the top right of the figure) and difference in ice thickness between 3D and rigid simulations (right, with corresponding color bar on the bottom right of the figure) in 2500. Green and black contours correspond to the grounding line position with the 3D and rigid Earth models, respectively.

The contribution of the West Antarctic Ice Sheet (WAIS) and associated local bedrock elevation changes to global mean sea-level change (ΔGMSL) for the full range of simulations are shown in Fig. 3 and table S1. With 3D viscoelastic Earth structure, under the RCP2.6-MISI scenario, the WAIS is projected to contribute 0.21 m by 2150 and 0.70 m by the end of the simulation at 2500. The contribution increases with higher emission scenarios, reaching 1.03 m for RCP4.5-MISI and 3.05 m for RCP8.5-MISI by 2500. When hydrofracture and ice cliffs are accounted for (i.e., with MICI), as in (*15*), the AIS contribution to ΔGMSL increases substantially, especially after the 21st century (Fig. 3). By 2500, the ΔGMSL contribution from WAIS reaches 1.68 m under RCP2.6-MICI, 2.15 m under RCP4.5-MICI, and 5.75 m under RCP8.5-MICI. The ice loss in all scenarios largely comes from the WAIS, whereas the EAIS experiences a small ice gain due to increased accumulation (fig. S2), except for RCP8.5-MICI in which EAIS ice loss is activated and the contribution from the whole ice sheet exceeds 15 m by 2500.

**Fig. 4.**
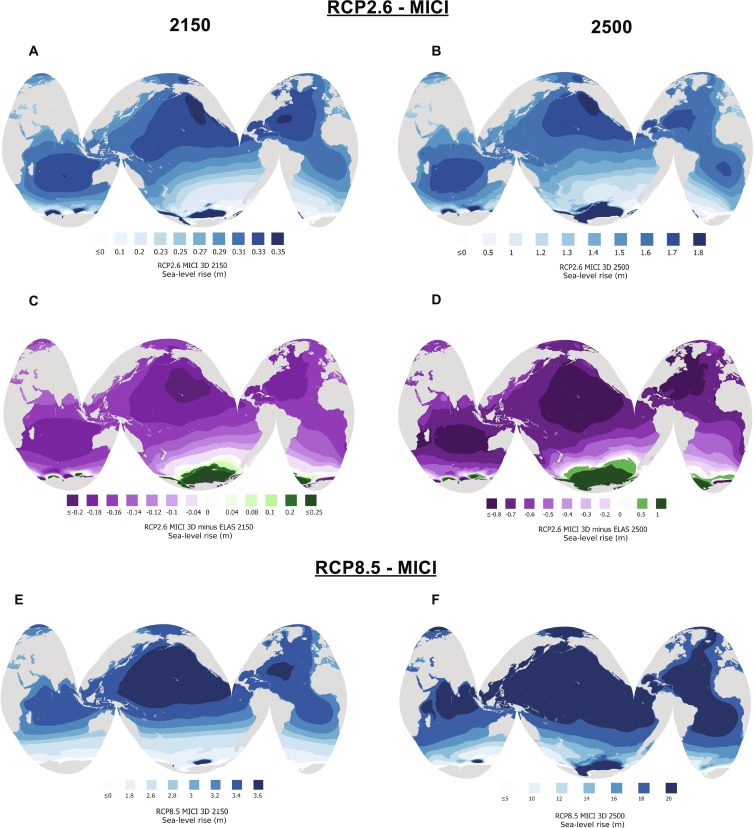
Global, spatially variable sea-level changes due to Antarctic ice loss. (**A** and **B**) Sea-level change in meters from (A) 2000 to 2150 and (B) 2000 to 2500 associated with ice cover changes across Antarctica for the RCP2.6-MICI scenario, computed with the SEAKON GIA model that includes GRD changes adopting the 3D viscoelastic Earth model shown in Fig. 2. (**C** and **D**) Difference between sea-level changes over the same periods as frames (A) and (B) computed with 3D viscoelastic and elastic Earth models. The result shows that viscous deformation associated with 3D Earth structure reduces global sea-level changes along most of the global coastline. (**E** and **F**) As in (A) and (B) but for the RCP8.5-MICI scenario, where 3D viscoelastic deformation increases projected far-field sea-level changes.

The projected Antarctic ice loss in all scenarios produces a geographically and temporally variable pattern of global sea-level change due to gravitational, Earth rotational, and deformational (GRD) effects that amplify sea level rise along low to mid latitude coastlines (Fig. 4). For example, the ΔGMSL contribution for the whole AIS (including WAIS mass loss and small EAIS mass gain) under RCP2.6-MICI is 0.27 m by 2150 and 1.46 m by 2500, while peaks of up to 0.33 and 1.8 m occur in the Pacific and Indian Oceans at these times when accounting for GRD effects. A similar pattern of amplification occurs across the rest of the simulations, except for RCP8.5-MICI in which both EAIS and WAIS on either side of the rotational pole undergo ice loss, reducing Earth rotational effects (*45*) and bringing the regions of peak sea-level rise closer to the equator (Fig. 4F). Note that all simulations produce higher than average sea-level rise due to Antarctic ice loss throughout the next 500 years for all Small Island Developing States (also called Large Ocean States), in agreement with previous studies (*46*, *47*). This geographic vulnerability will be further amplified due to higher-than-average sea-level rise associated with Greenland ice loss [e.g., (*47*)].

### Sensitivity of projected Antarctic sea-level contribution to Earth deformation and climate scenario

Comparing simulations adopting a range of different Earth structure models in Fig. 3, we find that GIA begins to influence the contribution of the WAIS to ΔGMSL on multi-century timescales for all climate and ice physics scenarios considered, starting as early as the end of the current century for RCP2.6. Uplift of the solid Earth and gravitational drawdown of the sea surface have the strongest effect when 3D viscoelastic Earth structure is adopted, where zones of thinned lithosphere and low mantle viscosity (Fig. 2) show faster, more localized bedrock uplift beneath areas of active WAIS marine ice retreat (fig. S6). With the 3D viscoelastic Earth structure, GIA reduces the WAIS contribution to ΔGMSL in all scenarios except RCP8.5-MICI. Differences between simulations with GIA and 3D Earth structure and those with fixed bedrock elevation (i.e., labeled “rigid”; from stand-alone ice-sheet model simulations; see Materials and Methods) reach over 50% during the simulations and are up to 40% for RCP2.6 and up to 25% for RCP4.5 cumulatively by 2500 (Fig. 3 and table S1A). Assuming a purely elastic Earth structure or a reference 1D viscoelastic model representative of continental average structure (see Materials and Methods and Fig. 2) neglects/underestimates the growing viscous uplift in low-viscosity zones, thereby weakening the sea-level feedback on ice dynamics and overestimating the WAIS contribution to ΔGMSL. ΔGMSL contribution is up to 34% and 17% higher with elastic and 1D viscoelastic Earth structures, respectively, relative to 3D Earth structure simulations.

The impact of GIA and 3D Earth structure on the ice sheet is larger and plays a role on ice dynamics sooner for lower emission scenarios. This is especially evident with MICI included, whereby the inclusion of 3D viscoelastic relative to rigid Earth structure reduces Antarctica’s GMSL contribution by >50% with RCP2.6-MICI, and up to 25% with RCP4.5-MICI (table S1). Under RCP8.5, grounding line retreat outpaces bedrock uplift, with retreat across WAIS marine sectors occurring within the first two centuries regardless of the adopted Earth model. However, after ice has completely retreated from WAIS marine basins, solid Earth uplift continues (fig. S4), driving water expulsion and increasing the WAIS contribution to ΔGMSL by 15% (0.8 m) by 2500 relative to the simulation with a rigid bed (Fig. 3F). In summary, we find that GIA effects reduce the WAIS contribution to GMSL rise under lower emissions through the sea-level feedback on marine ice-sheet grounding line dynamics (*18*), but amplify it under high-emission scenarios through water expulsion (Figs. 1 and 3) (*43*–*45*).

### Influence of Earth deformation on grounding line dynamics

**Fig. 5.**
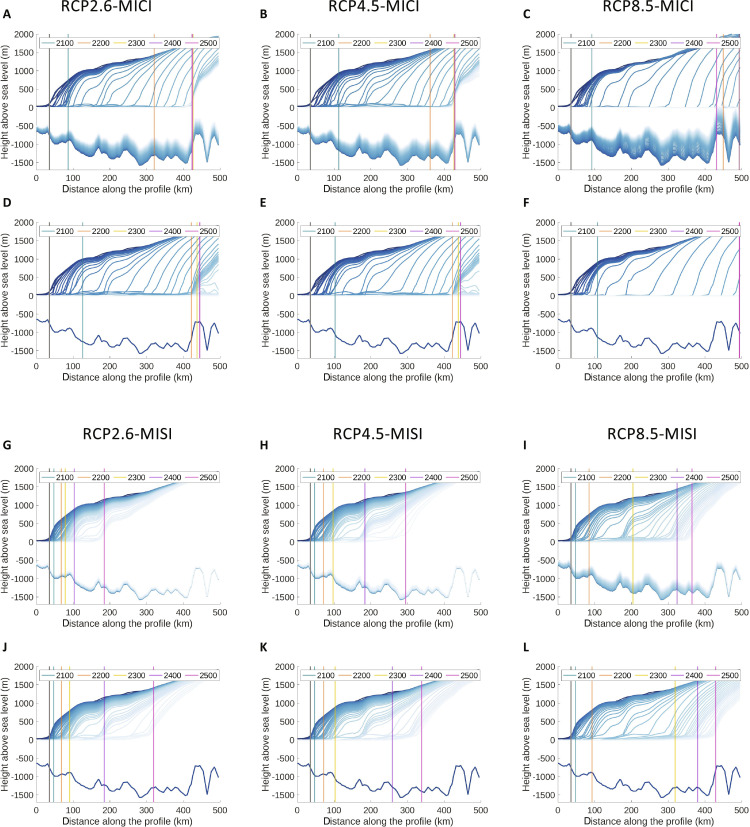
Impact of solid Earth rheology on grounding line migration in the Thwaites Glacier basin in nested ice-sheet model simulations. (**A** to **L**) Cross section along Thwaites Glacier (location shown in Fig. 2A) indicating changes in the ice surface elevation and bedrock elevation associated with all modeled climate and ice physics scenarios as labeled. As discussed in the text, MICI indicates that marine ice cliff physics and ice shelf hydrofracturing are included in the ice model, while MISI simulations exclude these physics and consider only marine ice-sheet physics. Simulations adopt either (A to C and G to I) the 3D viscoelastic Earth structure shown in Fig. 2 or (D to F and J to L) a rigid (i.e., nondeforming) Earth. Contours are plotted at 10-year time intervals from dark to light blue, with faster ice loss and bedrock uplift indicated by greater spacing between contours. Colored vertical lines mark grounding line positions at 2100, 2200, 2300, 2400, and 2500 in the simulations. The gray vertical lines indicate the initial 1950 grounding line position.

The impact of the sea-level feedback on the grounding line for a given ice physics and climate forcing scenario varies spatially and temporally between peripheral glaciers (e.g., compare the retreat of Thwaites and Pine Island glaciers in Fig. 5 and movies S1 to S8). Ice flow and grounding line migration depend on regional bedrock and ice geometry, including local bedrock elevation changes beneath retreating grounding lines. In all simulations, especially those with no MICI, grounding line retreat rates are variable rather than monotonic, accelerating when backing onto steep reversed slopes, and slowing or pausing when reaching bumps and pinning points (illustrated by grounding line positions in Fig. 5 and movies S1 to S8). With the deformable bedrock, the grounding line remains on bedrock highs longer, slowing the retreat compared to simulations with a rigid Earth. For example, with RCP2.6-MISI, the Thwaites grounding line retreats 134 km less by 2500 than with the rigid bed.

Consistent with previous models (*15*), invoking MICI results in faster and more extensive Thwaites grounding line retreat for a given warming scenario compared to MISI-only scenarios. When GIA is included in lower emission scenarios with MICI, accelerated rates of ice loss at the grounding line begin later and remain lower compared to rigid-bed simulations (fig. S3). Uplift of interior bedrock stabilizes the grounding line sooner than when the bed remains at its initial elevation. In RCP2.6-MICI with rigid bed, the grounding line retreats 40 km further inland by 2100, and 100 km further inland by 2200 compared to the simulation with 3D deformation. By 2300, the grounding line has retreated across most of the region in both simulations, but the retreat stops sooner on a bedrock high located ~425 km inland from the initial grounding line with deformation included, while the grounding line continues to retreat beyond this high when bedrock uplift is neglected (Fig. 5, A and D, and fig. S3). While extensive ice loss under RCP8.5-MICI leads to greater bedrock uplift than in other scenarios (figs. S4 to S6), the ice cliff retreat outpaces uplift, and most of the additional viscous uplift occurs after the grounding line has passed through the region, causing water expulsion and enhancing ΔGMSL (Fig. 4I and fig. S6D).

### Impact of viscous deformation on bedrock elevation and sea-level change at the grounding line and globally

Viscous effects contribute substantially to bedrock uplift and relative sea-level fall in Antarctica on decadal to centennial timescales (fig. S6). In simulations adopting a purely elastic Earth structure, peak uplift in the Amundsen Sea Embayment reaches 5 to 7 m by 2150 without MICI and 24 to 74 m with MICI. Peak deformation increases by 20 to 26 m without MICI and by 51 to 268 m with MICI at the same time points in simulations with 3D viscoelastic Earth structure (fig. S6). In scenarios with less/slower ice loss (e.g., fig. S6, A and C), substantially more uplift occurs with the 3D viscoelastic model compared to the elastic model during the period when the grounding line retreats across the basin. Conversely, with MICI and stronger climate forcing (fig. S6D), much of the grounding line retreat across the basin occurs before differences in the amount of uplift emerge between the different Earth models. Under RCP2.6 and with 3D viscous effects included, sea-level rise is up to 0.20 m lower in 2150 and 0.80 m lower in 2500 along global coastlines away from Antarctica (Fig. 4, C and D) relative to the Elastic Earth model or reference 1D viscoelastic Earth model (fig. S7).

**Fig. 6.**
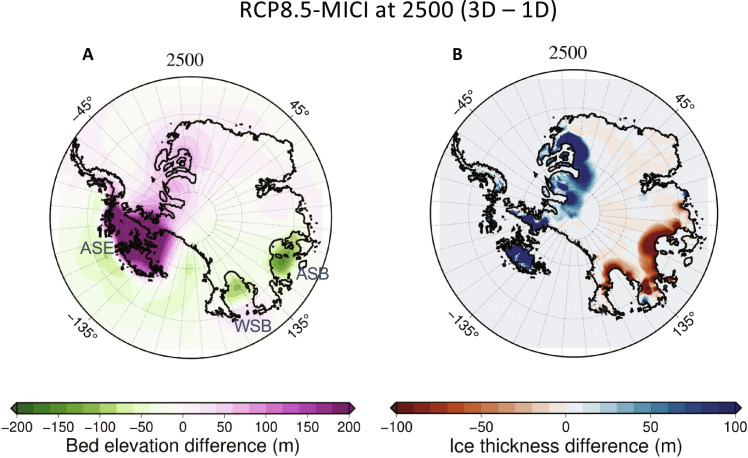
Impact of 3D Earth structure on predicted bedrock elevation and ice-sheet changes in continent-wide ice-sheet model simulations. (**A**) The difference in bed elevation change and (**B**) ice thickness between simulations adopting the 3D viscoelastic Earth structure versus reference (average) 1D viscoelastic structure at 2500 for scenario RCP8.5-MICI, which includes full collapse of marine ice sectors in West Antarctica and substantial retreat of ice in East Antarctica. Gray letters indicate the locations of the Amundsen Sea Embayment (ASE) and the Wilkes (WSB) and Aurora (ASB) subglacial basins.

Our results highlight the importance of considering lateral variability in Earth structure (Fig. 2A) in Antarctic-wide simulations (Fig. 6). With RCP8.5-MICI, substantial ice loss occurs in both East and West Antarctica. In West Antarctica, and especially the Amundsen Sea Embayment, low viscosities lead to greater uplift (Fig. 6A), reducing ice loss (Fig. 6B) compared to the simulation adopting an average radially varying Earth structure. In East Antarctica, higher than average mantle viscosities lead to less uplift beneath marine sectors, allowing for more ice loss compared to the average model. In particular, the bed remaining at lower elevations in the Wilkes and Aurora Subglacial Basins with the 3D Earth model allow for increased ice loss in these regions. The effects of this East to West contrast have also been explored in (*23*). Thus, purely radially varying Earth structure models do not accurately capture the deformation of the Earth and impact on the ice sheet in either East or West Antarctica.

### Importance of the method of computing ice-sheet contribution to ΔGMSL

In agreement with recent studies (*48*, *49*), the method adopted to compute ice-sheet ΔGMSL contribution affects the degree of influence of GIA on sea-level projections. Here, we use a method based on total water conservation (see Materials and Methods) that accounts for the effects of ice loss and water expulsion. This method produces the same results as the “modified volume-above-floatation (VAF)” method defined in recent studies (*48*, *50*, *51*), correcting an important calculation flaw in the basic VAF method widely used in earlier literature.

Differences in ΔGMSL due to the method chosen are discussed in the Supplementary Materials. Figure S1 shows ΔGMSL projections using the basic VAF method, which differ substantially from those in Fig. 3 (except in the rigid simulation), especially early in the simulations. Other differences later in the simulations are due to the water expulsion effect (*43*, *44*), which is included in our method but not in basic VAF or (*49*) (see Supplementary Materials). Caution should thus be taken when comparing calculations of ice-sheet ΔGMSL contributions in the literature, and it is important to explicitly state the method adopted in a given study.

## DISCUSSION

Our results indicate that GIA drives two competing effects that alter the AIS contribution to global sea levels: (i) the sea-level feedback on grounding line dynamics (*18*, *19*), in which solid Earth uplift and sea-level fall reduces grounding line retreat in marine basins in Antarctica, and (ii) the water expulsion effect (*43*, *44*, *48*), in which uplifting marine sectors freed of ice expel water out of Antarctica, increasing sea-level rise (Fig. 1). Here, we examine these effects together under a range of climate forcing and ice physics scenarios. When 3D variations in viscoelastic Earth structure across Antarctica are included in coupled ice sheet–GIA model simulations for the future, the first effect dominates in low-emission ice loss scenarios. Under RCP2.6, the sea-level feedback can reduce grounding line retreat by Ο(100 km) and reduce the contribution of the AIS to global sea-level rise by up to ~40% starting around 2100. These findings are consistent with regional studies and simplified treatments of GIA (*23*–*26*), finding that rapid viscous deformation has the potential to reduce retreat of individual glaciers in the Amundsen Sea Embayment on centennial timescales. For high-end projections under RCP8.5, the rapid ice-sheet retreat across marine sectors outpaces GIA and the ice-sheet dynamics are relatively insensitive to the choice of Earth model. In this case, water expulsion is the dominant effect, increasing global sea-level rise on multi-century timescales. Our findings suggest that sea-level physics should be accounted for in future generations of ice-sheet model projections informing Intergovernmental Panel on Climate Change Assessment Report 7 (IPCC-AR7) (*1*, *14*).

Uncertainty in the AIS’s contribution to future global sea-level projections remains substantial, with recent estimates ranging from minimal change to tens of meters of change on multi-century timescales. Uncertainties mainly stem from the choice of climate forcing (*52*, *53*), ice-sheet model differences (*14*), and Earth system feedbacks such as the solid Earth–ice interactions that are the focus here. The goal of our study is not to update future sea-level projections but rather to understand the influence of GIA feedbacks across a range of ice-sheet retreat timescales. Our work shows that GIA effects and the 3D rheological structure of the solid Earth play important roles in governing the response of the AIS to a warming climate with implications for impacts on global coastlines in the coming centuries.

Viscous deformation in our simulations takes place on decadal timescales in WAIS marine sectors (fig. S6), exceeding the contribution from elastic deformation to sea-level fall (bedrock elevation increase) near grounding lines and reaching tens to >100 m of uplift by 2150. We find that neglecting lateral heterogeneity in Earth structure results in substantive bias in the amplitude and spatial patterns of the projected ice loss, deformation, and projected global sea level in continent-wide simulations (Fig. 6 and fig. S7). Future work is needed to clarify to what degree radially varying Earth structure models can be applied to represent deformation and ice-Earth interactions accurately across regional and basin scales, and ambiguity remains on how best to define an optimally applicable regional 1D Earth model. Alternative inferences of 1D Earth structure to approximate GIA across regions of laterally heterogeneous Earth structure, such as West Antarctica, yield substantial differences depending on the methodology adopted and the observations considered (*26*, *54*). Coupled ice sheet–GIA model projections adopting different estimates of 1D WAIS structure from the literature [extended data figure 8 from (*15*)] can produce different levels of grounding line stabilization. Recent seismic surface wave tomography suggests that there is heterogeneity in Earth structure on <100-km spatial scales in the Amundsen Sea Embayment (*55*) that can produce notable differences in predicted GIA at basin scale modern and future grounding lines relative to continental 3D and 1D Earth models (*56*). Details of the local bedrock setting also matter, with spatiotemporally variable retreat and ice cliff calving rates depending on the detailed bed geometry (Fig. 5 and fig. S4). Comparison of ice-Earth interactions at different glaciers from our results (movies S1 to S8) suggests that the degree of applicability of 1D Earth models may be basin specific.

Our findings suggest that further constraints on Earth structure from improved seismic imaging and bedrock elevation changes from ongoing geodetic (GNSS) observations will be needed to provide further insight on this matter. Improved length and spatial coverage of observations will be important for both reducing uncertainty in ice-sheet projections and improving the interpretation of geophysical and geological observations in Antarctica. Achieving better resolution of Earth structure in coastal West Antarctica where seismic observations are sparse, and particularly in areas of active ice loss in the Amundsen Sea Embayment region, is critical to understanding of grounding line retreat patterns.

Sea-level changes along coastlines in our simulations are spatially variable, peaking in the Caribbean, along the western coast of Africa and in the Pacific and Indian Ocean basins (Fig. 4). In many of these regions, when accounting for 3D viscoelastic Earth structure globally, sea-level rise due to Antarctic ice loss reaches up to 1.7 m for low-end projections under RCP2.6 and up to 19.5 m by 2500 for high-end projections. Our results further support recent findings (*46*, *47*) that low-latitude islands and coastal sites already being affected by sea-level rise will experience higher than average sea-level rise associated with Antarctic ice loss, regardless of the ice loss scenario. This finding highlights the climate injustice toward nations whose emissions are low, while their exposure and vulnerability to sea-level rise is high (*46*). We have found that the effect of GIA is to reduce the Antarctic contribution to global sea-level rise for low emissions and amplify it for high emissions. Therefore, reducing greenhouse gas emissions will allow the rebound of the solid Earth to play a greater role in preserving more of the AIS and avoiding the worst and most inequitable impacts of future climate change on global coastlines.

## MATERIALS AND METHODS

### Experimental design

We use an ice sheet–sea-level coupling approach detailed and applied to the last deglaciation in (*42*) and adapted to future simulations here to model AIS thickness change and global GIA (i.e., including bedrock elevation changes relative to the geoid beneath the AIS, and gravitationally consistent global sea-level change) for a period of 550 years, from 1950 to 2500. In this approach, the PSUICE3D ice-sheet/shelf model (*15*, *57*) is coupled to the SEAKON 3D, global GIA model (*41*, *42*) that incorporates deformation of a range of 3D and 1D Earth structure models. We describe the two models and the coupling procedure below, along with the adopted Earth structure models below.

### Ice-sheet model

We simulate AIS dynamics using the PSUICE3D model (*58*, *59*). The model uses a heuristic combination of shallow-ice and shallow-shelf approximations according to model equations and specifications described in detail in (*58*, *59*), with climate forcing and setup for future simulations described in (*15*). Key elements of the model setup, climate forcing, and details of the coupling procedure are described below. The simulations are run to 2500 as described in (*15*), following the extended RCP scenarios provided by (*60*).

The ice simulations are performed in two different modes. First, an initial continental-wide run with 10-km resolution is performed followed by a nested 5-km resolution simulation over West Antarctica (Fig. 1, boundary of the nested domain is marked with a black box). The latter resolution is the finest feasible over such a large area, and nested tests over the Amundsen Sea Embayment in (*15*) showed minimal resolution dependency with grid spacing ranging from 1 to 10 km. Both continental and nested modes start from a modern state in 1950 and extend until 2500, with 1-year time stepping. The continental simulations provide the necessary lateral boundary conditions for ice thickness and velocity in the nested model runs. Basal sliding coefficients are derived from surface velocities using the inverse methodology outlined in (*61*). Bathymetry and ice surface elevation at the start of the simulations are taken from Bedmap2 (*62*) [note that ice model tests not shown here show only minor differences between simulations adopting Bedmap2 and BedMachine (*4*) bedrock topography in these types of large-scale runs]. Atmospheric and oceanic forcing for the ice-sheet model are provided as described in (*15*). Annual surface mass balance is calculated from monthly mean precipitation and surface air temperatures in a regional climate model (*63*), adapted to Antarctica. Meteorological climate forcing and subsurface temperatures determining sub–ice-shelf melt rates in the model follows three future Representative Concentration Pathway (RCP) scenarios: RCP2.6, RCP4.5, and RCP8.5 (*60*). For consistency with previous work, ice-sheet initial conditions (ice thickness, bed elevation, velocity, basal sliding coefficients, and internal ice and bed temperatures) follow the same procedure described (*15*), with a 100,000-year spin-up using observed climate forcing. Ice-sheet initial conditions are identical in all simulations.

Marine ice-sheet instability (MISI) dynamics are captured in all simulations. Half of the simulations incorporate the effects of hydrofracturing and mechanical failure of marine-terminating ice cliffs (MICI) as described in (*15*). These later simulations rely on two key, adjustable cliff-calving parameters (one controlling the ice thickness penetrated by hydrofracturing and the other controlling the maximum horizontal ice wastage rate of the cliff face), which we select based on the average calibrated values that are best able to reproduce Last Interglacial, Pliocene, and modern observations of ice loss as in (*15*).

### 3D global GIA model

To compute global sea-level changes and evolution of bedrock elevation beneath the AIS, we adopt a 3D, finite volume GIA model (*41*) that solves a generalized form of the sea-level equation that is described in detail (*45*), accounting for time-varying migration of shorelines (*64*, *65*), load-induced changes in Earth rotation (*66*), and the viscoelastic deformation of a self-gravitating, elastically compressible Maxwell (viscoelastic) Earth with 3D rheological structure. As input to the 3D GIA model, spatiotemporal evolution of grounded ice is provided by the ice-sheet model (see Materials and Methods) and a range of different rheological structures of the Earth are adopted, as discussed below.

Computations are performed on a global tetrahedral grid with triangulated spherical surfaces, and the model includes grid-refinement capabilities to achieve higher resolution across regions of West Antarctica characterized by active ice loss and low viscosity [see the supplementary materials from (*42*)]. Results of sensitivity experiments in our previous work from (*38*) indicate that GIA predictions associated with modern and future ice loss converge for surface resolutions finer than 4 km, with small differences for surface resolutions less than 15 km, with the limiting factor the ability of the grid to capture the ice loading changes. On the basis of these findings, our computational grid reaches ~3-km surface resolution over the refined area indicated by the green contour in Fig. 2, ~7-km surface resolution over the rest of the Antarctic continent, and 12 to 15 km over the rest of the globe. Lateral resolution decreases with depth to ~50 km at the core-mantle boundary. Radially, there are 67 spherical layers spaced at similar distances to the lateral resolution of the corresponding triangulated surface to ensure relatively regular geometry for the tetrahedrons. The resulting grid is composed of ~28 million grid nodes and ~160 million elements.

### Earth structure

We consider purely elastic (green lines in Fig. 3, labeled ELAS), radially varying (1D) viscoelastic and 3D viscoelastic models of Earth structure in our simulations. We also perform “rigid” simulations (blue lines in Fig. 3, labeled RIGID) with the ice-sheet model alone, where bedrock and sea surface elevations remain fixed in time.

As in (*38*), elastic and density structure in all GIA model simulations is radially varying and based on the seismic model STW105 (*40*). The reference 1D viscoelastic Earth model (adopted in simulations represented by orange lines in Fig. 3, labeled 1D) is characterized by uniform viscosities of 10^20^ Pa s in the upper mantle (from the bottom of the lithosphere to the depth of 670 km) and 5 × 10^21^ Pa s in the lower mantle (from 670-km depth to the core-mantle boundary) and a lithospheric thickness of 96 km, representing the average thickness across Antarctica in the 3D viscoelastic models described below. The mantle viscosities in the 1D model serve as the reference profile for perturbations in viscosities in the 3D viscosity model described below.

The 3D viscoelastic model we adopt (shown in Fig. 2, and adopted in simulations shown by red lines in Fig. 3, labeled 3D) is first derived and described in detail in (*38*) (see description of model EM3D_L). Viscosity variations relative to the 1D reference model described above are based on the ANT-20 seismic shear-wave speed model of the upper mantle described in (*31*) beneath Antarctica and the S362ANI seismic tomography model (*40*) across the rest of the globe. The S362ANI model has a resolution of 𝒪(1000 km). The ANT-20 model resolves structures with a spatial scale of ~100 km down to 410-km depth and higher at greater depths. Variations in lithospheric thickness are based on the model of (*67*) globally and the model of (*35*) over Antarctica. As described in (*68*), the lithospheric thickness model is scaled to an average of 96 km over Antarctica, reaching a minimum thickness of 40 km in Marie Byrd Land.

Seismic velocities from the global and regional Antarctic tomography models are converted to viscosity variations following the procedure described in (*41*) (see their equations 27 and 29), by first converting seismic velocity anomalies into temperature anomalies, and adopting a scaling factor, ϵ, to relate temperature to viscosity. A scaling factor of 0.033°C^−1^ is adopted in Antarctica (ANT-20) and 0.04°C^−1^ for the global model (S362ANI) based on comparison to GNSS inferences of vertical viscosity structure beneath the Amundsen Sea Embayment (*26*), Fleming Glacier in central Antarctic Peninsula (*29*), and northern Antarctic Peninsula (*28*). More detail on this procedure is provided in (*38*).

### Coupled ice sheet–3D GIA model simulations

We perform a suite of coupled ice sheet–global GIA model simulations, repeating results with the range of different adopted models of Earth structure described above, under extended RCP emission trajectories RCP2.6, RCP4.5, and RCP8.5 (*60*), both with and without MICI-related processes included. Note that sea-level changes due to Greenland and mountain glacier ice loss and thermosteric effects are not included in our model and will also alter sea levels around Antarctica. However, these contributions will be small at marine grounding lines compared to the local drawdown of the sea surface and uplift of the solid Earth associated with local ice loss (*68*, *69*).

The coupling procedure is as follows. First, the ice thickness changes across the simulation time from 1950 to 2500 are calculated from continental and nested simulations with the ice-sheet model alone with 1-year time stepping (see above). Then, the results are combined, with nested simulations defining WAIS ice cover changes and continental runs predicting ice loss over the rest of Antarctica, and passed as ice loading input for the 3D GIA model. The GIA model then computes the global GIA changes based on these ice cover changes at 2-year time increments across the simulation time, and these results are linearly interpolated to 1-year time increments. The 2-year time increments in the GIA model substantially reduce computation time, and initial testing of this procedure showed negligible differences between computing GIA every year, and every 2 years and then linearly interpolating. Changes in sea level (i.e., changes in elevation of the geoid relative to the solid Earth surface) are calculated globally and passed to the ice-sheet model over the ice-sheet model domain, where they are then used to provide changes in bed elevation every year in a new ice-sheet simulation. We repeat the coupling process three to four times to allow for convergence of predicted ice volume changes, following findings on convergence in (*42*). Figure S1 from (*42*) demonstrates convergence with our method for paleo-simulations and shows that results with the iterative method are comparable to those produced with the standard interactive coupling procedure from earlier work with a radially varying GIA model (*70*). Note that while these tests suggest that our coupling procedure is appropriate for capturing the continental-scale ice-GIA interactions that are the focus of this study, further testing may be necessary for future applications focusing on more localized ice cover changes.

### Global mean sea-level calculations

Estimates of the contribution of the AIS to global mean sea-level change (ΔGMSL), shown in Fig. 3, are calculated from the ice-sheet model results using a method based on the conservation of global water mass. It is appropriate in simulations with no major changes in ice sheets or shorelines in the rest of the world. Neglecting relatively small changes in other reservoirs such as groundwater (*71*), changes in total ocean, and ice mass within the Antarctic ice-sheet model domain (*ANT*) are equal in magnitude and of opposite sign to those in the rest of the world (*REST*). Also neglecting steric changes (*71*), this implies

$$\text{ΔGMSL}\times A_{\text{RO}}=-\sum_{\text{ANT}}∆\left(\frac{\rho_{i}}{\rho_{w}}h+\text{max}\left[G-h_{b}-\frac{\rho_{i}}{\rho_{w}}h,0\right]\right)$$ (1)

where *A*_*RO*_ is the modern oceanic area outside the Antarctic ice model domain (*REST*) and ΔGMSL is the increase in sea level, i.e., ocean column thickness or the equivalent where there is floating ice, averaged over *REST*. The left-hand side with invariant *A*_*RO*_ assumes that any changes in shorelines and ice distribution within *REST* are negligible. ΔGMSL is taken to be the Antarctic “contribution” to global sea-level change, because in our simulations there are no prescribed ice changes in the rest of the world. Σ is the sum over all grid cells in the relevant Antarctic region *ANT* (a weighting term equal to grid-cell area is omitted for clarity in all summations here). The region ANT can be limited to West or East Antarctic sectors (separated by the pink line in Fig. 2A). Δ indicates the change from initial modern to current time. *h* is ice thickness (grounded or floating), *G* is the elevation of the geoid (coinciding with the sea surface where no floating ice), and *h*_b_ is bedrock elevation, both relative to a fixed Earth reference level. The term in square brackets is the column thickness of liquid ocean water, equal to the radial distance from the seafloor to the base of floating ice, or to the sea surface if no ice. ρ_i_ = 910 kg m^−3^ is ice density and ρ_w_ = 1028 kg m^−3^ is ocean water density.

Equation 1 yields exactly the same results as the “modified VAF” method recommended, for instance, in (*48*)

$$\begin{array}{c} \Delta \mathrm{GMSL}\prime \times A_{\text{RO}}=\\ -\sum_{\;\text{ANT}}∆\left(\text{max}\;\left[\frac{\rho_{i}}{\rho_{w}}h-\text{max}\left[G-h_{b},0\right],0\right]+\text{max}\left[G-h_{b},0\right]\right) \end{array}$$ (2)

Equation 2 is equivalent to that used in (*48*, *50*, *72*), except for small “max” corrections that are needed for generality. The expressions in large parentheses on the right-hand sides of Eqs. 1 and 2, i.e., the total water equivalent volume and the modified volume over floatation for individual grid points, are exactly equal for all points (terrestrial or marine, grounded, or floating ice). For grounded ice points [where (ρ_i_/ρ_w_) *h* > *G* − *h*_b_], both are equal to (ρ_i_/ρ_w_)*h*. For floating ice points or open ocean, both are equal to *G* − *h*_b_. For land points with no ice, both are zero. Therefore, the summed totals over ANT in each equation are equal.

For reference, the method using “basic” VAF is

$$\begin{array}{c} \Delta \mathrm{GMSL}\prime \prime \times \left(A_{\text{RO}}+A_{\text{AO}}\right)=\\ -\sum_{\;\text{ANT}}∆\{\text{max}\;\left[\frac{\rho_{i}}{\rho_{w}}h-\text{max}\left[G-h_{b},0\right],0\right]\} \end{array}$$ (3)

which is just Eq. 2 without the additional bathymetric term (+ max [*G* − *h*_b_, 0]) on the right-hand side. This calculation is applied to the global ocean area, i.e., *A*_*RO*_* + A*_*AO*_ on the left-hand side (where *A*_*AO*_ is the oceanic area in the Antarctic domain, using its modern value as in previous studies). The basic VAF method has been used as a standard in many previous publications. However, recent studies (*48*, *49*) show that without the bathymetric term, the basic VAF method is flawed and produces ΔGMSL estimates substantially different from the more accurate methods. The main flaw is that if marine ice columns remain grounded, changes in bedrock elevation with constant ice thickness cause basic VAF to change when there should be no effect on sea level.

For comparison, fig. S1 shows ΔGMSL’s for our simulations calculated using basic VAF. As expected, there are notable differences from Fig. 3. Differences between simulations adopting different Earth models generally emerge sooner and are larger when using the basic VAF method. Sensitivity tests show that this is nearly all due to the flaw in basic VAF described above. Therefore, early in our simulations before large grounding line retreat, changes in bedrock elevation below grounded marine ice spuriously affect ΔGMSL projections in basic VAF (fig. S1) but not in our results (Fig. 3).

Because our method includes the bathymetric term for all points including open ocean and floating ice, there is another difference from basic VAF: The “water expulsion” effect is included. This effect is produced by rising or falling bedrock elevations under open ocean or floating ice within the Antarctic domain, which displaces water into or out of the rest of the world oceans (*43*, *44*). The impact of water expulsion is particularly pronounced for RCP8.5-MICI with Earth deformation, causing ΔGMSL projections to be higher than those with rigid beds after ~2200 (Fig. 3C). In contrast, using basic VAF (without water expulsion), the ΔGMSL projections for RCP8.5-MICI with Earth deformation remain lower than those with rigid beds (fig. S1C).

Adhikari *et al*. (*49*) also correct the main flaw in basic VAF for ice that remains grounded, effectively in the same way as above (in their equation 11). However, they do not include the bathymetric term for open ocean or floating ice so that water expulsion is excluded from their calculation and their ΔGMSL estimate is an average for the entire world ocean area. This is consistent with the observation that the net effect of water expulsion on global mean sea level is zero, because it only redistributes water regionally and does not change global oceanic (plus floating ice) mass. The effects of water expulsion in Fig. 3 can be isolated by subtracting the ΔGMSL values from those using the method of (*49*) (not shown). The differences are small compared to the total in most cases [consistent with (*44*)], except for the later stages of RCP8.5-MICI with deforming Earth when water expulsion is pronounced, as mentioned above.

Whether or not to include the water expulsion effect in ΔGMSL calculations may depend on the focus of a particular study. We include it to assess the impact of changes in Antarctic ice, solid Earth, and sea levels on the rest of the world’s coastlines; however, excluding the effect may be more appropriate for other applications, such as ice-sheet model intercomparison efforts, and to normalize predictions of spatially variable impacts of gravitational, rotational, and deformational (GRD) effects on global ocean depths. Regardless, it is important to explicitly state the method adopted in a given study.

Note that for the simulations with rigid beds (black curves), the ΔGMSL estimates for corresponding simulations in Fig. 3 and fig. S1 are nearly identical, i.e., the basic VAF method yields essentially the same results as the more accurate methods. That is because the bathymetric correction term needed to convert “basic” to “modified” VAF (+ max [*G* − *h*_b_, 0]) is invariant in time for simulations in which bedrock and geoid elevations are held constant; therefore, the ΔGMSL estimates with basic VAF are the same as with modified VAF and our calculations. In other words, the main flaw inherent in basic VAF does not arise if bedrock and sea-surface elevations remain constant [also noted by (*49*)]. Furthermore, the water expulsion mechanism is not operative in this case. [There are still slight differences between the rigid-bed curves in Fig. 3 versus fig. S1, because the resetting of sea-surface elevations in the simulations themselves is unphysical and does not conserve global water mass; i.e., the changes in sea level *G* − *h*_b_ in Eq. 1 and the last term in Eq. 2 for Antarctic oceanic areas are (unphysically) zero, and so do not reduce the meltwater supplied to the rest of the world as they should, causing a slight overestimate of ΔGMSL in those equations.]

Although not shown in Eqs. 1 to 3 above, all ΔGMSL calculations here include a “density correction” that accounts for the difference between fresh meltwater and ocean water (*48*, *49*), which amounts to subtracting the total change in solid ice volume (floating or grounded) multiplied by ρ_i_/ρ_f_ − ρ_i_/ρ_w_ from the right-hand sides, where ρ_f_ = meltwater density 1000 kg m^−3^. This correction is minor, ~5% or less of ΔGMSL and generally uniform across simulations, in the type of future Antarctic simulations performed here [sensitivity tests not shown, see also (*48*)].

## References

[1] Intergovernmental Panel on Climate Change, Climate Change 2021 – The Physical Science Basis. Contribution of Working Group I to the Sixth Assessment Report of the Intergovernmental Panel on Climate Change. V. Masson-Delmotte, P. Zhai, A. Pirani, S. L. Connors, C. Péan, S. Berger, N. Caud, Y. Chen, L. Goldfarb, M. I. Gomis, M. Huang, K. Leitzell, E. Lonnoy, J. B. R. Matthews, T. K. Maycock, T. Waterfield, O. Yelekçi, R. Yu, B. Zhou, Eds. (Cambridge Univ. Press, 2021); 10.1017/9781009157896.

[2] E. Kirezci, I. R. Young, R. Ranasinghe, S. Muis, R. J. Nicholls, D. Lincke, J. Hinkel, Projections of global-scale extreme sea levels and resulting episodic coastal flooding over the 21st century. Sci. Rep. 10, 11629 (2020). 32732976 10.1038/s41598-020-67736-6 PMC7393110

[3] T. L. Noble, E. J. Rohling, A. R. A. Aitken, H. C. Bostock, Z. Chase, N. Gomez, L. M. Jong, M. A. King, A. N. Mackintosh, F. S. M. Cormack, R. M. M. Kay, L. Menviel, S. J. Phipps, M. E. Weber, C. J. Fogwill, B. Gayen, N. R. Golledge, D. E. Gwyther, A. M. C. Hogg, Y. M. Martos, B. Pena-Molino, J. Roberts, T. van de Flierdt, T. Williams, The sensitivity of the Antarctic ice sheet to a changing climate: Past, present, and future. Rev. Geophys. 58, e2019RG000663 (2020).

[4] M. Morlighem, E. Rignot, T. Binder, D. Blankenship, R. Drews, G. Eagles, O. Eisen, F. Ferraccioli, R. Forsberg, P. Fretwell, V. Goel, J. S. Greenbaum, H. Gudmundsson, J. Guo, V. Helm, C. Hofstede, I. Howat, A. Humbert, W. Jokat, N. B. Karlsson, W. S. Lee, K. Matsuoka, R. Millan, J. Mouginot, J. Paden, F. Pattyn, J. Roberts, S. Rosier, A. Ruppel, H. Seroussi, E. C. Smith, D. Steinhage, B. Sun, M. R. van den Broeke, T. D. van Ommen, M. van Wessem, D. A. Young, Deep glacial troughs and stabilizing ridges unveiled beneath the margins of the Antarctic ice sheet. Nat. Geosci. 13, 132–137 (2020).

[5] S. L. Cornford, D. F. Martin, A. J. Payne, E. G. Ng, A. M. Le Brocq, R. M. Gladstone, T. L. Edwards, S. R. Shannon, C. Agosta, M. R. van den Broeke, H. H. Hellmer, G. Krinner, S. R. M. Ligtenberg, R. Timmermann, D. G. Vaughan, Century-scale simulations of the response of the West Antarctic Ice Sheet to a warming climate. Cryosphere 9, 1579–1600 (2015).

[6] I. Joughin, B. E. Smith, B. Medley, Marine Ice Sheet Collapse Potentially Under Way for the Thwaites Glacier Basin, West Antarctica. Science 344, 735–738 (2014). 24821948 10.1126/science.1249055

[7] B. R. Parizek, K. Christianson, S. Anandakrishnan, R. B. Alley, R. T. Walker, R. A. Edwards, D. S. Wolfe, G. T. Bertini, S. K. Rinehart, R. A. Bindschadler, S. M. J. Nowicki, Dynamic (in)stability of Thwaites Glacier, West Antarctica. J. Geophys. Res. Earth 118, 638–655 (2013).

[8] D. Pollard, R. M. DeConto, Modelling West Antarctic ice sheet growth and collapse through the past five million years. Nature 458, 329–332 (2009). 19295608 10.1038/nature07809

[9] C. Schoof, Ice sheet grounding line dynamics: Steady states, stability, and hysteresis. J. Geophys. Res. 112, 227–252 (2007).

[10] J. Weertman, Stability of the junction of an ice sheet and an ice shelf. J. Glaciol. 13, 3–11 (1974).

[11] The IMBIE team, Mass balance of the Antarctic Ice Sheet from 1992 to 2017. Nature 558, 219–222 (2018). 29899482 10.1038/s41586-018-0179-y

[12] G. R. Grant, T. R. Naish, G. B. Dunbar, P. Stocchi, M. A. Kominz, P. J. J. Kamp, C. A. Tapia, R. M. M. Kay, R. H. Levy, M. O. Patterson, The amplitude and origin of sea-level variability during the Pliocene epoch. Nature 574, 237–241 (2019). 31578526 10.1038/s41586-019-1619-z

[13] A. Dutton, A. E. Carlson, A. J. Long, G. A. Milne, P. U. Clark, R. DeConto, B. P. Horton, S. Rahmstorf, M. E. Raymo, Sea-level rise due to polar ice-sheet mass loss during past warm periods. Science 349, aaa4019 (2015). 26160951 10.1126/science.aaa4019

[14] H. Seroussi, S. Nowicki, A. J. Payne, H. Goelzer, W. H. Lipscomb, A. Abe-Ouchi, C. Agosta, T. Albrecht, X. Asay-Davis, A. Barthel, R. Calov, R. Cullather, C. Dumas, B. K. Galton-Fenzi, R. Gladstone, N. R. Golledge, J. M. Gregory, R. Greve, T. Hattermann, M. J. Hoffman, A. Humbert, P. Huybrechts, N. C. Jourdain, T. Kleiner, E. Larour, G. R. Leguy, D. P. Lowry, C. M. Little, M. Morlighem, F. Pattyn, T. Pelle, S. F. Price, A. Quiquet, R. Reese, N.-J. Schlegel, A. Shepherd, E. Simon, R. S. Smith, F. Straneo, S. Sun, L. D. Trusel, J. Van Breedam, R. S. W. van de Wal, R. Winkelmann, C. Zhao, T. Zhang, T. Zwinger, ISMIP6 Antarctica: A multi-model ensemble of the Antarctic ice sheet evolution over the 21st century. Cryosphere 14, 3033–3070 (2020).

[15] R. M. De Conto, D. Pollard, R. B. Alley, I. Velicogna, E. Gasson, N. Gomez, S. Sadai, A. Condron, D. M. Gilford, E. L. Ashe, R. E. Kopp, D. Li, A. Dutton, The Paris Climate Agreement and future sea-level rise from Antarctica. Nature 593, 83–89 (2021). 33953408 10.1038/s41586-021-03427-0

[16] D. M. Gilford, E. L. Ashe, R. M. DeConto, R. E. Kopp, D. Pollard, A. Rovere, Could the last interglacial constrain projections of future Antarctic ice mass loss and sea-level rise? J. Geophys. Res. Earth 125, e2019JF005418 (2020).

[17] A. A. Robel, H. Seroussi, G. H. Roe, Marine ice sheet instability amplifies and skews uncertainty in projections of future sea-level rise. Proc. Natl. Acad. Sci. U.S.A. 116, 14887–14892 (2019). 31285345 10.1073/pnas.1904822116 PMC6660720

[18] N. Gomez, J. X. Mitrovica, P. Huybers, P. U. Clark, Sea level as a stabilizing factor for marine-ice-sheet grounding lines. Nat. Geosci. 3, 850–853 (2010).

[19] N. Gomez, D. Pollard, J. X. Mitrovica, P. Huybers, P. U. Clark, Evolution of a coupled marine ice sheet-sea level model. J. Geophys. Res. Earth 117, F01013 (2012).

[20] N. Gomez, D. Pollard, D. Holland, Sea-level feedback lowers projections of future Antarctic Ice-Sheet mass loss. Nat. Commun. 6, 8798 (2015). 26554381 10.1038/ncomms9798 PMC5426515

[21] H. Konrad, I. Sasgen, D. Pollard, V. Klemann, Potential of the solid-Earth response for limiting long-term West Antarctic Ice Sheet retreat in a warming climate. Earth Planet. Sci. Lett. 432, 254–264 (2015).

[22] E. Larour, H. Seroussi, S. Adhikari, E. Ivins, L. Caron, M. Morlighem, N. Schlegel, Slowdown in Antarctic mass loss from solid Earth and sea-level feedbacks. Science 364, eaav7908 (2019). 31023893 10.1126/science.aav7908

[23] V. Coulon, K. Bulthuis, P. L. Whitehouse, S. Sun, K. Haubner, L. Zipf, F. Pattyn, Contrasting response of West and East Antarctic ice sheets to glacial isostatic adjustment. J. Geophys. Res. Earth 126, e2020JF006003 (2021).

[24] C. Book, M. J. Hoffman, S. B. Kachuck, T. R. Hillebrand, S. F. Price, M. Perego, J. N. Bassis, Stabilizing effect of bedrock uplift on retreat of Thwaites Glacier, Antarctica, at centennial timescales. Earth Planet. Sci. Lett. 597, 117798 (2022).

[25] S. B. Kachuck, D. F. Martin, J. N. Bassis, S. F. Price, Rapid viscoelastic deformation slows marine ice sheet instability at Pine Island Glacier. Geophys. Res. Lett. 47, e2019GL086446 (2020).

[26] V. R. Barletta, M. Bevis, B. E. Smith, T. Wilson, A. Brown, A. Bordoni, M. Willis, S. A. Khan, M. Rovira-Navarro, I. Dalziel, R. Smalley, E. Kendrick, S. Konfal, D. J. Caccamise, R. C. Aster, A. Nyblade, D. A. Wiens, Observed rapid bedrock uplift in Amundsen Sea Embayment promotes ice-sheet stability. Science 360, 1335–1339 (2018). 29930133 10.1126/science.aao1447

[27] G. A. Nield, P. L. Whitehouse, W. van der Wal, B. Blank, J. P. O’Donnell, G. W. Stuart, The impact of lateral variations in lithospheric thickness on glacial isostatic adjustment in West Antarctica. Geophys. J. Int. 214, 811–824 (2018).

[28] G. A. Nield, V. R. Barletta, A. Bordoni, M. A. King, P. L. Whitehouse, P. J. Clarke, E. Domack, T. A. Scambos, E. Berthier, Rapid bedrock uplift in the Antarctic Peninsula explained by viscoelastic response to recent ice unloading. Earth Planet. Sci. Lett. 397, 32–41 (2014).

[29] C. Zhao, M. A. King, C. S. Watson, V. R. Barletta, A. Bordoni, M. Dell, P. L. Whitehouse, Rapid ice unloading in the Fleming Glacier region, southern Antarctic Peninsula, and its effect on bedrock uplift rates. Earth Planet. Sci. Lett. 473, 164–176 (2017).

[30] W. Shen, D. A. Wiens, S. Anandakrishnan, R. C. Aster, P. Gerstoft, P. D. Bromirski, S. E. Hansen, I. W. D. Dalziel, D. S. Heeszel, A. D. Huerta, A. A. Nyblade, R. Stephen, T. J. Wilson, J. P. Winberry, The crust and upper mantle structure of central and West Antarctica from Bayesian inversion of Rayleigh wave and receiver functions. J. Geophys. Res. Solid Earth 123, 7824–7849 (2018).

[31] A. J. Lloyd, D. A. Wiens, H. Zhu, J. Tromp, A. A. Nyblade, R. C. Aster, S. E. Hansen, I. W. Dalziel, T. J. Wilson, E. R. Ivins, Seismic structure of the Antarctic upper mantle imaged with adjoint tomography. J. Geophys. Res. Solid Earth 125, (2020).

[32] S. E. Hansen, J. H. Graw, L. M. Kenyon, A. A. Nyblade, D. A. Wiens, R. C. Aster, A. D. Huerta, S. Anandakrishnan, T. Wilson, Imaging the Antarctic mantle using adaptively parameterized P-wave tomography: Evidence for heterogeneous structure beneath West Antarctica. Earth Planet. Sci. Lett. 408, 66–78 (2014).

[33] W. R. Peltier, D. Argus, R. Drummond, Space geodesy constrains ice age terminal deglaciation: The global ICE-6G_C (VM5a) model. J. Geophys. Res. Solid Earth 120, 450–487 (2015).

[34] P. L. Whitehouse, M. J. Bentley, G. A. Milne, M. A. King, I. D. Thomas, A new glacial isostatic adjustment model for Antarctica: Calibrated and tested using observations of relative sea-level change and present-day uplift rates. Geophys. J. Int. 190, 1464–1482 (2012).

[35] M. An, M. An, D. A. Wiens, Y. Zhao, D. A. Wiens, M. Feng, Y. Zhao, A. Nyblade, M. Feng, M. Kanao, Y. Li, A. Maggi, J. J. Lévêque, Temperature, lithosphere-asthenosphere boundary, and heat flux beneath the Antarctic Plate inferred from seismic velocities. J. Geophys. Res. Solid Earth 120, 8720–8742 (2015).

[36] S. D. Boger, Antarctica—Before and after Gondwana. Gondw. Res. 19, 335–371 (2011).

[37] T. A. Jordan, T. R. Riley, C. S. Siddoway, The geological history and evolution of West Antarctica. Nat. Rev. Earth Environ. 1, 117–133 (2020).

[38] J. X. W. Wan, N. Gomez, K. Latychev, H. K. Han, Resolving glacial isostatic adjustment (GIA) in response to modern and future ice loss at marine grounding lines in West Antarctica. Cryosphere 16, 2203–2223 (2022).

[39] D. Pollard, N. Gomez, R. M. Deconto, Variations of the Antarctic Ice Sheet in a coupled ice sheet-Earth-sea level model: Sensitivity to viscoelastic Earth properties. J. Geophys. Res. Earth 122, 2124–2138 (2017).

[40] B. Kustowski, G. Ekström, A. M. Dziewoński, Anisotropic shear-wave velocity structure of the Earth’s mantle: A global model. J. Geophys. Res. Solid Earth 113, (2008).

[41] K. Latychev, J. X. Mitrovica, J. Tromp, M. E. Tamisiea, D. Komatitsch, C. C. Christara, Glacial isostatic adjustment on 3-D Earth models: A finite-volume formulation. Geophys. J. Int. 161, 421–444 (2005).

[42] N. Gomez, K. Latychev, D. Pollard, A coupled ice sheet-sea level model incorporating 3D Earth structure: Variations in Antarctica during the last deglacial retreat. J. Clim. 31, 4041–4054 (2018).

[43] L. Pan, E. M. Powell, K. Latychev, J. X. Mitrovica, J. R. Creveling, N. Gomez, M. J. Hoggard, P. U. Clark, Rapid postglacial rebound amplifies global sea level rise following West Antarctic Ice Sheet collapse. Sci. Adv. 7, eabf7787 (2021). 33931453 10.1126/sciadv.abf7787 PMC8087405

[44] M. Yousefi, J. Wan, L. Pan, N. Gomez, K. Latychev, J. Mitrovica, D. Pollard, R. DeConto, The influence of the solid Earth on the contribution of marine sections of the Antarctic Ice Sheet to future sea-level change. Geophys. Res. Lett. 49, e2021GL097525 (2022).

[45] N. Gomez, J. X. Mitrovica, M. E. Tamisiea, P. U. Clark, A new projection of sea level change in response to collapse of marine sectors of the Antarctic Ice Sheet. Geophys. J. Int. 180, 623–634 (2010).

[46] S. Sadai, R. Spector, R. DeConto, N. Gomez, The Paris Agreement and climate justice: Inequitable impacts of sea level rise associated with temperature targets. Earths Future 10, e2022EF002940 (2022).

[47] J. Roffman, N. Gomez, M. Yousefi, H. K. Han, S. Nowicki, Spatial and temporal variability of 21st century sea level changes. Geophys. J. Int. 235, 342–352 (2023).

[48] H. Goelzer, V. Coulon, F. Pattyn, B. De Boer, R. Van De Wal, Brief communication: On calculating the sea-level contribution in marine ice-sheet models. Cryosphere 14, 833–840 (2020).

[49] S. Adhikari, E. R. Ivins, E. Larour, L. Caron, H. Seroussi, A kinematic formalism for tracking ice–ocean mass exchange on the earth’s surface and estimating sea-level change. Cryosphere 14, 2819–2833 (2020).

[50] A. M. Dolan, B. De Boer, J. Bernales, D. J. Hill, A. M. Haywood, High climate model dependency of Pliocene Antarctic ice-sheet predictions. Nat. Commun. 9, 2799 (2018). 30022077 10.1038/s41467-018-05179-4 PMC6052068

[51] B. de Boer, A. M. Dolan, J. Bernales, E. Gasson, H. Goelzer, N. R. Golledge, J. Sutter, P. Huybrechts, G. Lohmann, I. Rogozhina, A. Abe-Ouchi, F. Saito, R. S. W. van de Wal, Simulating the Antarctic ice sheet in the late-Pliocene warm period: PLISMIP-ANT, an ice-sheet model intercomparison project. Cryosphere 9, 881–903 (2015).

[52] A. Barthel, C. Agosta, C. M. Little, T. Hattermann, N. C. Jourdain, H. Goelzer, S. Nowicki, H. Seroussi, F. Straneo, T. J. Bracegirdle, CMIP5 model selection for ISMIP6 ice sheet model forcing: Greenland and Antarctica. Cryosphere 14, 855–879 (2020).

[53] D. Li, R. M. DeConto, D. Pollard, Climate model differences contribute deep uncertainty in future Antarctic ice loss. Sci. Adv. 9, eadd7082 (2023). 36791186 10.1126/sciadv.add7082 PMC9931235

[54] E. Powell, K. Latychev, N. Gomez, J. Mitrovica, The robustness of geodetically derived 1-D Antarctic viscosity models in the presence of complex 3-D viscoelastic Earth structure. Geophys. J. Int. 231, 118–128 (2022).

[55] E. M. Lucas, A. A. Nyblade, A. J. Lloyd, R. C. Aster, D. A. Wiens, J. P. O’Donnell, G. W. Stuart, T. J. Wilson, I. W. D. Dalziel, J. P. Winberry, A. D. Huerta, Seismicity and Pn velocity structure of central West Antarctica. Geochem. Geophys. Geosyst. 22, e2020GC009471 (2021).

[56] E. Lucas, N. Gomez, K. Latychev, M. Yousefi, The impact of regional-scale variability in upper mantle viscosity on GIA in West Antarctica, EGU General Assembly 2024, Vienna, Austria, 14 to 19 April 2024, EGU24-13928; 10.5194/egusphere-egu24-13928.

[57] D. Pollard, R. M. DeConto, R. B. Alley, Potential Antarctic Ice Sheet retreat driven by hydrofracturing and ice cliff failure. Earth Planet. Sci. Lett. 412, 112–121 (2015).

[58] D. Pollard, R. M. DeConto, Description of a hybrid ice sheet-shelf model, and application to Antarctica. Geosci. Model Dev. 5, 1273–1295 (2012).

[59] D. Pollard, R. M. DeConto, Continuous simulations over the last 40 million years with a coupled Antarctic ice sheet-sediment model. Palaeogeogr. Palaeoclimatol. Palaeoecol. 537, 109374 (2020).

[60] M. Meinshausen, S. J. Smith, K. Calvin, J. S. Daniel, M. L. T. Kainuma, J.-F. Lamarque, K. Matsumoto, S. A. Montzka, S. C. B. Raper, K. Riahi, A. Thomson, G. J. M. Velders, D. P. P. van Vuuren, The RCP greenhouse gas concentrations and their extensions from 1765 to 2300. Clim. Change 109, 213–241 (2011).

[61] D. Pollard, R. M. DeConto, A simple inverse method for the distribution of basal sliding coefficients under ice sheets, applied to Antarctica. Cryosphere 6, 953–971 (2012).

[62] P. Fretwell, H. D. Pritchard, D. G. Vaughan, J. L. Bamber, N. E. Barrand, R. Bell, C. Bianchi, R. G. Bingham, D. D. Blankenship, G. Casassa, G. Catania, D. Callens, H. Conway, A. J. Cook, H. F. J. Corr, D. Damaske, V. Damm, F. Ferraccioli, R. Forsberg, S. Fujita, Y. Gim, P. Gogineni, J. A. Griggs, R. C. A. Hindmarsh, P. Holmlund, J. W. Holt, R. W. Jacobel, A. Jenkins, W. Jokat, T. Jordan, E. C. King, J. Kohler, W. Krabill, M. Riger-Kusk, K. A. Langley, G. Leitchenkov, C. Leuschen, B. P. Luyendyk, K. Matsuoka, J. Mouginot, F. O. Nitsche, Y. Nogi, O. A. Nost, S. V. Popov, E. Rignot, D. M. Rippin, A. Rivera, J. Roberts, N. Ross, M. J. Siegert, A. M. Smith, D. Steinhage, M. Studinger, B. Sun, B. K. Tinto, B. C. Welch, D. Wilson, D. A. Young, C. Xiangbin, A. Zirizzotti, Bedmap2: Improved ice bed, surface and thickness datasets for Antarctica. Cryosphere 7, 375–393 (2013).

[63] J. S. Pal, F. Giorgi, X. Bi, N. Elguindi, F. Solmon, X. Gao, S. A. Rauscher, R. Francisco, A. Zakey, J. Winter, M. Ashfaq, F. S. Syed, J. L. Bell, N. S. Diffenbaugh, J. Karmacharya, A. Konaré, D. Martinez, R. P. da Rocha, L. C. Sloan, A. L. Steiner, Regional climate modeling for the developing world: The ICTP RegCM3 and RegCNET. Bull. Am. Meteorol. Soc. 88, 1395–1410 (2007).

[64] R. A. Kendall, J. X. Mitrovica, G. A. Milne, On post-glacial sea level—II. Numerical formulation and comparative results on spherically symmetric models. Geophys. J. Int. 161, 679–706 (2005).

[65] J. X. Mitrovica, G. A. Milne, On post-glacial sea level: I. General theory. Geophys. J. Int. 154, 253–267 (2003).

[66] J. X. Mitrovica, J. Wahr, I. Matsuyama, A. Paulson, The rotational stability of an ice-age earth. Geophys. J. Int. 161, 491–506 (2005).

[67] C. P. Conrad, C. P. Conrad, C. Lithgow-Bertelloni, Influence of continental roots and asthenosphere on plate-mantle coupling. Geophys. Res. Lett. 33, L05312 (2006).

[68] C. C. Hay, H. C. P. Lau, N. Gomez, J. Austermann, E. Powell, J. X. Mitrovica, K. Latychev, D. A. Wiens, Sea level fingerprints in a region of complex earth structure: The case of WAIS. J. Clim. 30, 1881–1892 (2017).

[69] N. R. Golledge, E. D. Keller, N. Gomez, K. A. Naughten, J. Bernales, L. D. Trusel, T. L. Edwards, Global environmental consequences of twenty-first-century ice-sheet melt. Nature 566, 65–72 (2019). 30728520 10.1038/s41586-019-0889-9

[70] N. Gomez, D. Pollard, J. X. Mitrovica, A 3-D coupled ice sheet–sea level model applied to Antarctica through the last 40 ky. Earth Planet. Sci. Lett. 384, 88–99 (2013).

[71] A. R. Simms, L. Lisiecki, G. Gebbie, P. L. Whitehouse, J. F. Clark, Balancing the last glacial maximum (LGM) sea-level budget. Quat. Sci. Rev. 205, 143–153 (2019).

[72] B. de Boer, P. Stocchi, R. S. W. van de Wal, A fully coupled 3-D ice-sheet - sea-level model: Algorithm and applications. Geosci. Model Dev. 7, 2141–2156 (2014).

