# Supplementary material for: The influence of realistic 3D mantle viscosity on Antarctica’s contribution to future global sea levels
Source: Sci Adv. 2024 Aug 2;10(31):eadn1470. doi: 10.1126/sciadv.adn1470 (PMC11296330; doi:10.1126/sciadv.adn1470)
Supplement: Supplementary file 1 — Figs. S1 to S7 Table S1 Legends for movies S1 to S8 [file sciadv.adn1470_sm.pdf]

Science Advances
AAAS

Supplementary Materials for

The influence of realistic 3D mantle viscosity on Antarctica's contribution to future global sea levels

Natalya Gomez *et al.*

Corresponding author: Natalya Gomez, natalya.gomez@mcgill.ca

Sci. Adv. **10**, eadn1470 (2024)
DOI: 10.1126/sciadv.adn1470

The PDF file includes:

Figs. S1 to S7
Table S1
Legends for movies S1 to S8

Other Supplementary Material for this manuscript includes the following:

Movies S1 to S8

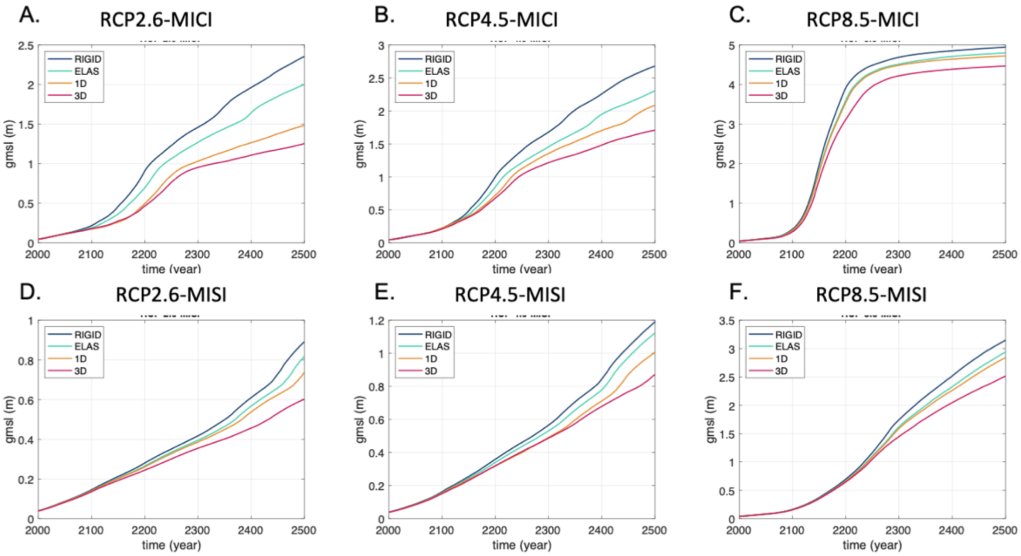

Fig. S1.

West Antarctic ice volume changes and contribution to global mean sea level change for a range of adopted Earth structure models, in nested ice-sheet model simulations, just for the region of West Antarctica indicated by the pink line in Fig. 1a of the main text. **(A- F)** as in Figure 2 of the main text, but adopting a basic volume-above-floatation (VAF) method to compute global mean sea level contribution, see Methods.

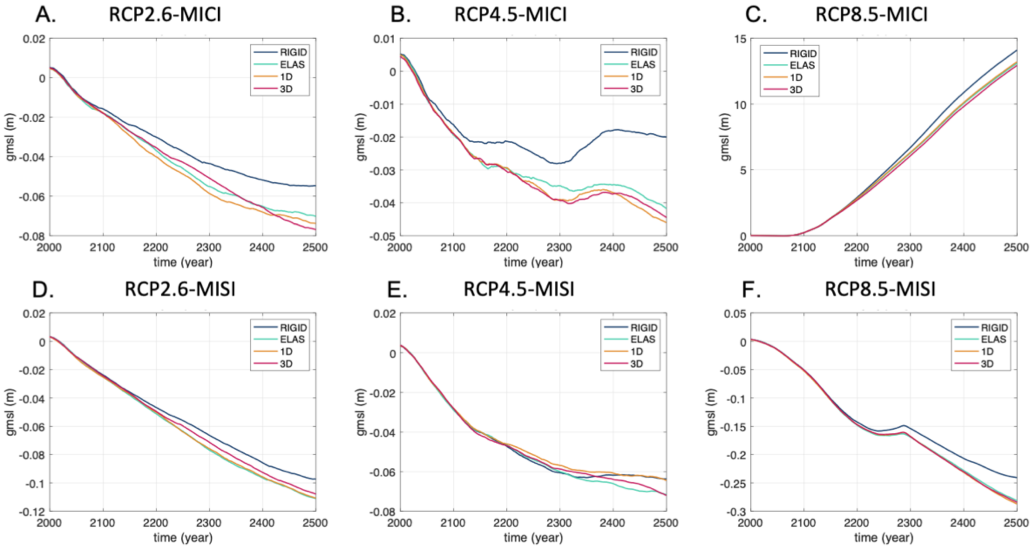

Fig. S2.

Contribution of the East Antarctic ice sheet to global mean sea level change under scenarios considered in Figure 2 of the main text, calculated from continent-wide 10km resolution ice sheet model simulations. The divide between East and West Antarctica in these calculations is shown by the pink line in Figure 1a of the main text.

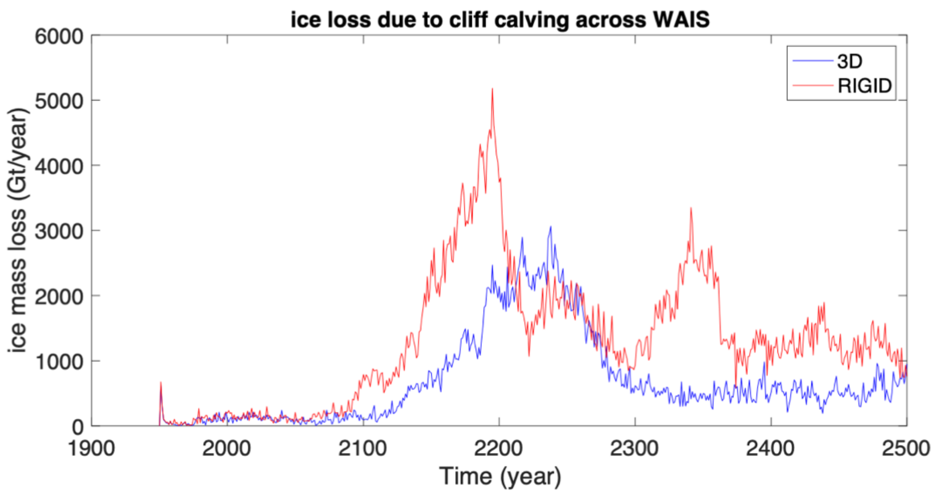

Fig. S3.
Impact of GIA on ice mass loss due to the marine ice cliff mechanism (MICI). Integrated rate of ice mass loss at the grounding line due to cliff calving across the whole nested WAIS simulation with RCP2.6-MICI scenario in Gt/year. The red line indicates the rate for the simulation adopting 3-D viscoelastic Earth structure while the blue line shows the rate with a rigid bed.

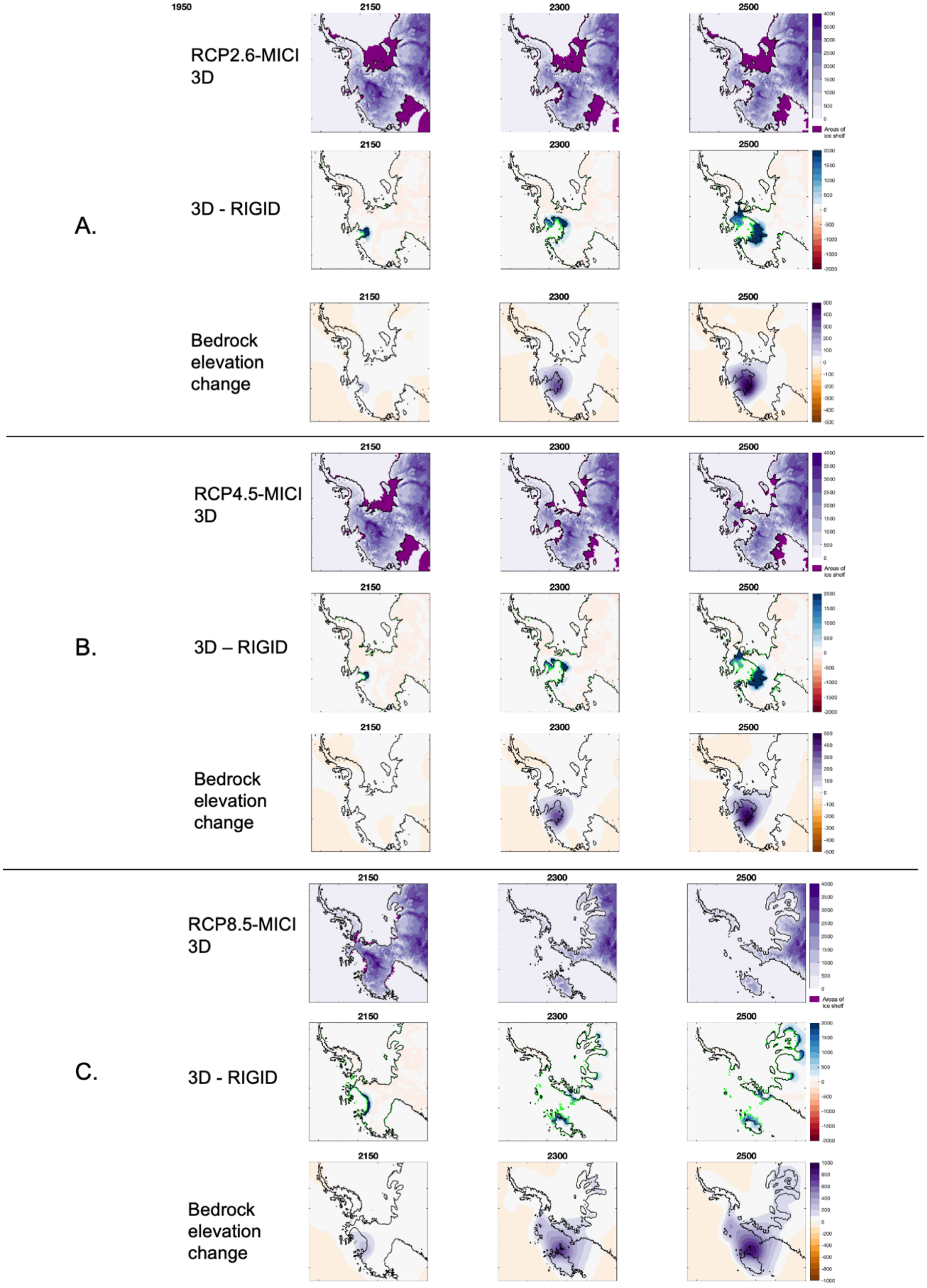
1950
2150
2300
2500
A.
RCP2.6-MICI 3D
3D - RIGID
Bedrock elevation change
Areas of ice shelf
B.
RCP4.5-MICI 3D
3D – RIGID
Bedrock elevation change
C.
RCP8.5-MICI 3D
3D - RIGID
Bedrock elevation change

Fig. S4.
Ice and bedrock evolution for simulations adopting MICI under RCP2.6 **(A)**, RCP4.5 **(B)** and RCP8.5 **(C)**. (top rows) Ice thickness from simulations with 3-D Earth structure, (middle rows) difference in ice thickness between 3-D and rigid simulations and (bottom rows) GIA (plotted as changes in elevation of the bedrock relative to the geoid) from the start of the simulations with 3-D Earth structure. All color scales are in meters.

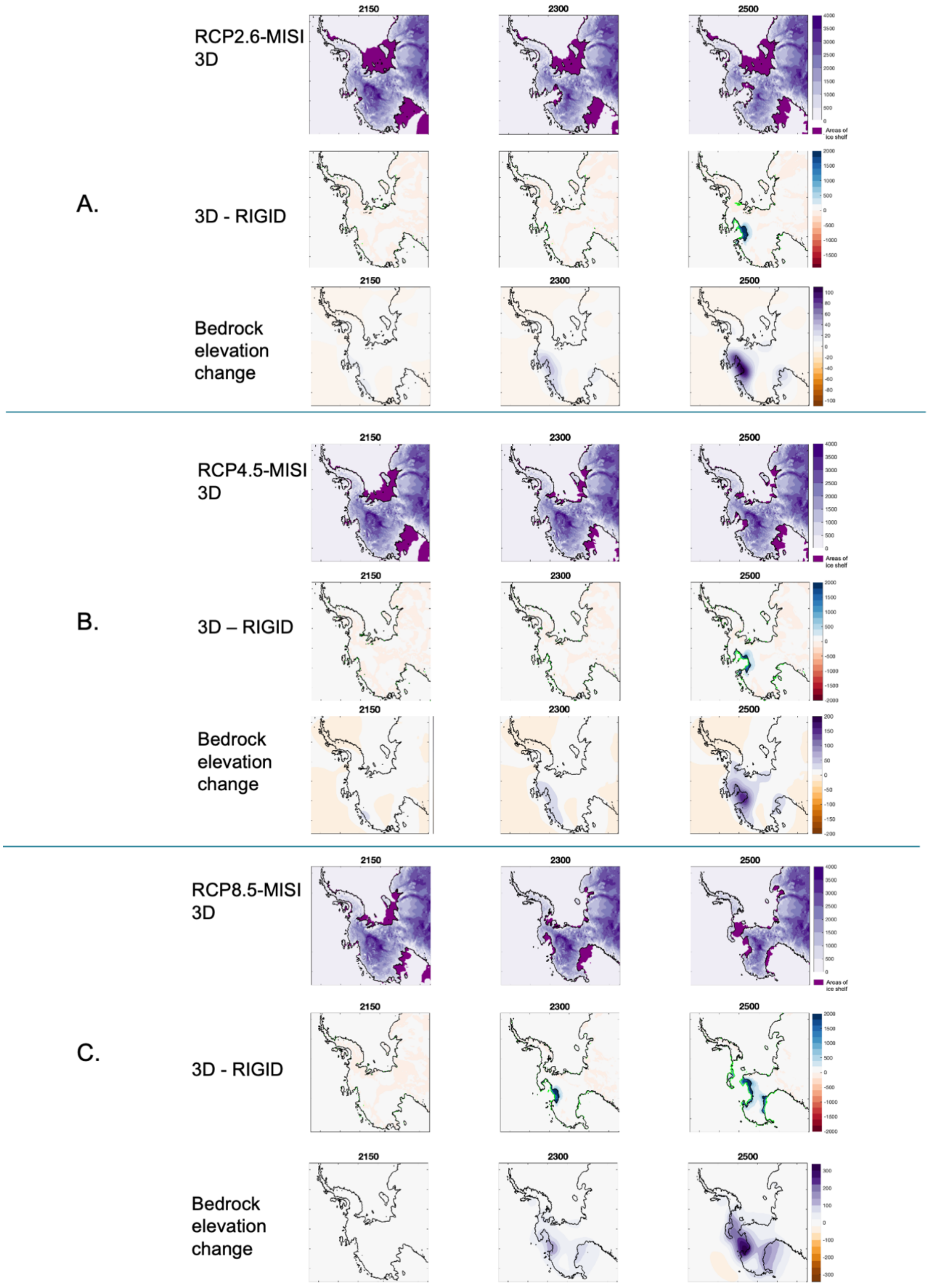

Fig. S5.
Ice and bedrock evolution for simulations without MICI under RCP2.6 **(A)**, RCP4.5 **(B)** and RCP8.5 **(C)**. (top rows) Ice thickness from simulations with 3-D Earth structure, (middle rows)

difference in ice thickness between 3-D and rigid simulations and (bottom rows) GIA (plotted as changes in elevation of the bedrock relative to the geoid) from the start of the simulations with 3-D Earth structure. All color scales are in meters. As in Supp. Fig. 4 but for MISI simulations.

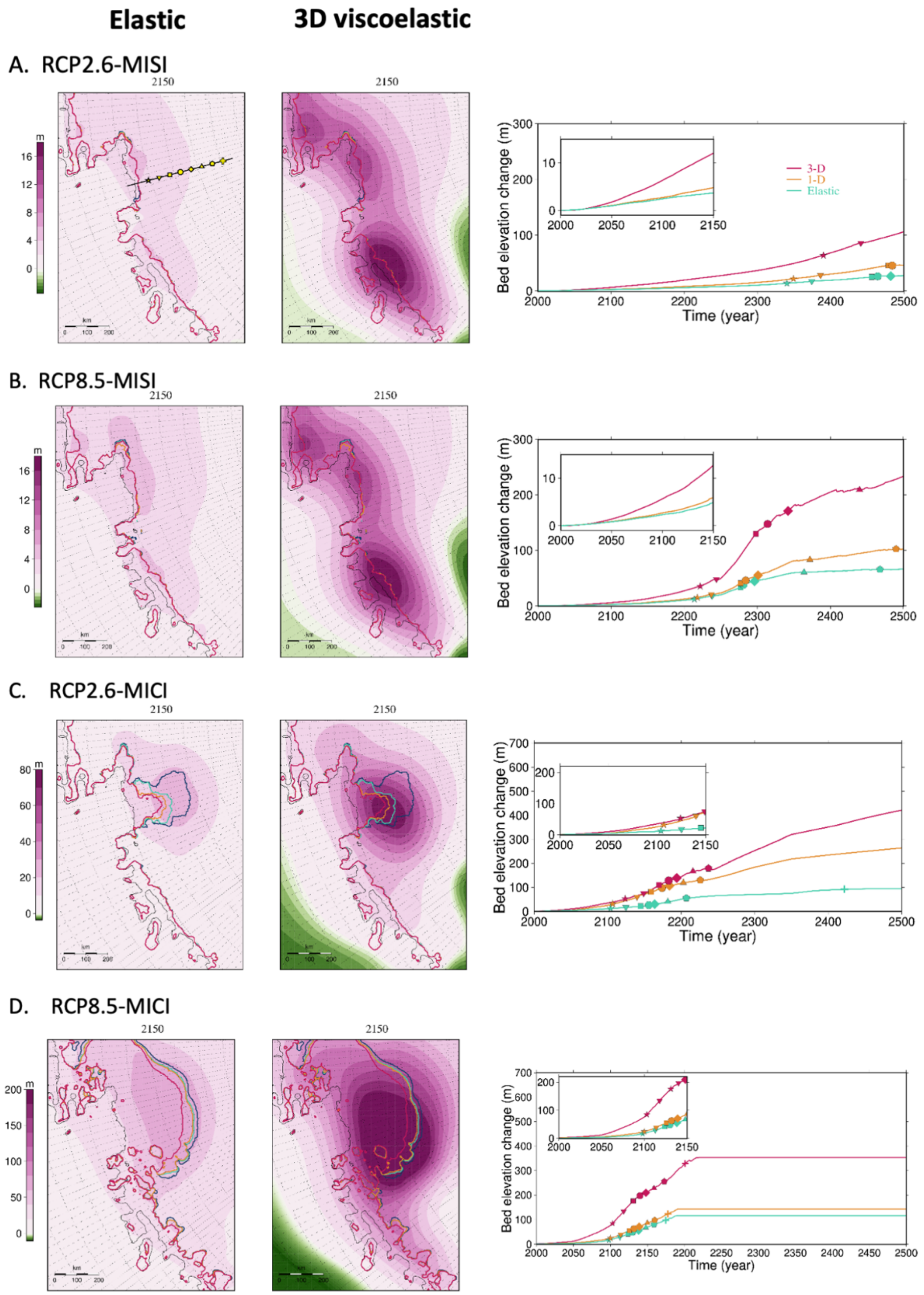

Fig. S6.

Elastic and viscous contributions to changes in bedrock elevation in the Amundsen Sea Embayment in nested ice-sheet model simulations. **(A-D)** The change in bedrock elevation at 2150 associated with the elastic Earth model (left panel) and the 3-D viscoelastic Earth model (middle panel), without **(A.B)** and with **(C, D)** MICI processes included, and associated with low

(A, C) and high-end **(B, D)** climate warming scenarios. Grounding line positions are plotted on each frame for different Earth structure assumptions with colors as in Figure 2: rigid Earth in dark blue, elastic model in green, spherically symmetric Earth model (1-D) in orange and 3-D model in red. Frames on the right represent bed elevation changes at the grounding line along the black transection shown in the left panel of frame (A) for the time period of 2000 to 2500. Several symbols are plotted along the lines to indicate the time when the grounding line position reaches locations along the cross section every hundred kilometers, starting with star symbols after 100 km of grounding line retreat. An inset for the model results until 2150 is also included. Note that in (D), the right plot only goes until 2200 since the grounding line retreats beyond the transect after this time.

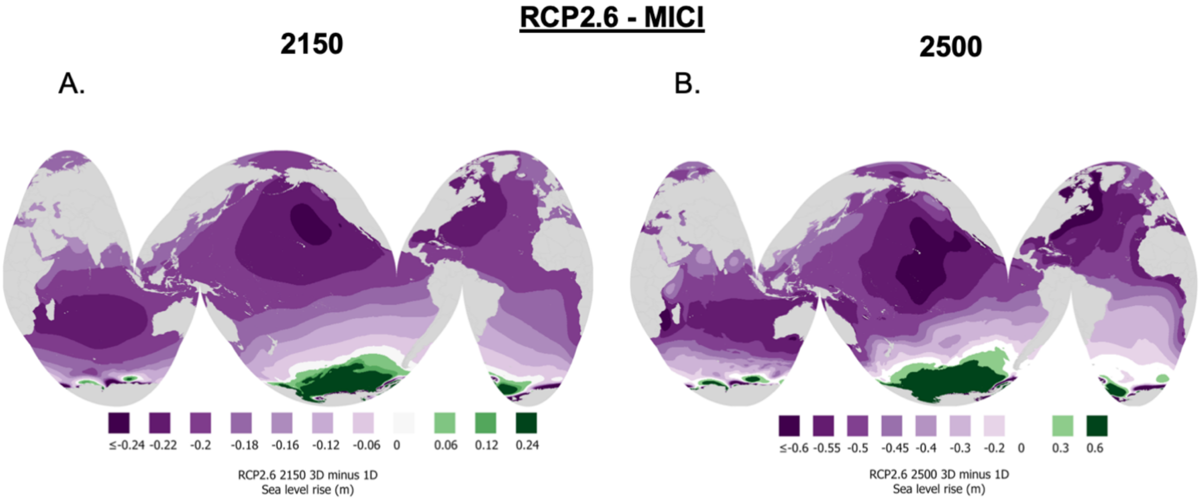

Fig. S7
The impact of laterally variable Earth structure on global, spatially variable sea-level changes due to Antarctic ice loss. (A-B) the difference between sea-level changes in meters from **(A)** 2000 to 2150 and **(B)** 2000 to 2500 computed with 3-D viscoelastic and 1-D viscoelastic reference models. As in Fig. 4(c-d) of the main text except comparing to a 1-D viscoelastic rather than elastic model.

A. **WAIS GMSL contribution in (m) as in Fig. 2**

RCP 2.6 - MICI

Year	3D	1D	Elastic	Rigid
2150	0.33	0.37	0.40	0.46
2300	1.23	1.32	1.43	1.46
2500	1.68	1.87	2.25	2.35

RCP 4.5 - MICI

Year	3D	1D	Elastic	Rigid
2150	0.43	0.47	0.50	0.48
2300	1.51	1.59	1.67	1.68
2500	2.15	2.48	2.62	2.68

RCP 8.5 - MICI

Year	3D	1D	Elastic	Rigid
2150	1.95	2.05	2.06	1.96
2300	5.30	5.22	5.16	4.69
2500	5.75	5.54	5.45	4.94

RCP 2.6 - MISI

Year	3D	1D	Elastic	Rigid
2150	0.21	0.22	0.22	0.21
2300	0.40	0.42	0.42	0.42
2500	0.70	0.82	0.88	0.89

RCP 4.5 - MISI

Year	3D	1D	Elastic	Rigid
2150	0.26	0.26	0.26	0.25
2300	0.55	0.55	0.57	0.56
2500	1.03	1.15	1.21	1.19

RCP 8.5 - MISI

Year	3D	1D	Elastic	Rigid
2150	0.38	0.38	0.38	0.37
2300	1.69	1.77	1.78	1.74
2500	3.05	3.21	3.23	3.15

B. **Impact of GIA with 3D viscoelastic Earth structure on WAIS GMSL contribution (in m and % difference - (3D-Rigid)/3D)**

	2100		2150		2200		2300		2400		2500	
Scenario	(m)	(%)	(m)	(%)	(m)	(%)	(m)	(%)	(m)	(%)	(m)	(%)
RCP 2.6 - MICI	-0.01	-7%	-0.14	-42%	-0.32	-55%	-0.23	-19%	-0.50	-34%	-0.67	-40%
RCP 4.5 - MICI	0.01	4%	-0.05	-12%	-0.20	-25%	-0.16	-11%	-0.39	-21%	-0.53	-25%
RCP 8.5 - MICI	0.00	1%	0.00	0%	-0.01	0%	0.61	12%	0.75	13%	0.80	14%
RCP 2.6 - MISI	0.00	1%	0.00	-1%	-0.01	-4%	-0.02	-5%	-0.08	-16%	-0.19	-28%
RCP 4.5 - MISI	0.00	2%	0.00	0%	0.00	-1%	-0.02	-3%	-0.07	-9%	-0.16	-15%
RCP 8.5 - MISI	0.00	2%	0.01	2%	0.01	2%	-0.05	-3%	-0.04	-2%	-0.10	-3%

C. **Impact of viscous effects with 3D Earth structure on WAIS GMSL contribution (in m and % difference - (3D-Elastic)/3D)**

Scenario	2100		2150		2200		2300		2400		2500	
	(m)	(%)	(m)	(%)	(m)	(%)	(m)	(%)	(m)	(%)	(m)	(%)
**RCP 2.6 - MICI**	-0.01	-3%	-0.08	-24%	-0.20	-35%	-0.20	-16%	-0.39	-26%	-0.57	-34%
**RCP 4.5 - MICI**	-0.01	-4%	-0.06	-14%	-0.19	-24%	-0.16	-10%	-0.35	-19%	-0.47	-22%
**RCP 8.5 - MICI**	-0.01	-2%	-0.11	-6%	-0.24	-6%	0.14	3%	0.24	4%	0.30	5%
**RCP 2.6 - MISI**	0.00	-1%	-0.01	-2%	-0.01	-4%	-0.02	-6%	-0.08	-16%	-0.18	-26%
**RCP 4.5 - MISI**	0.00	-1%	-0.01	-2%	-0.01	-2%	-0.02	-4%	-0.06	-8%	-0.18	-17%
**RCP 8.5 - MISI**	0.00	-1%	0.00	0%	0.00	0%	-0.09	-5%	-0.10	-4%	-0.19	-6%

Table S1.
Projected WAIS contribution to GMSL changes for the suite of simulations varying the adopted Earth structure model, RCP and ice physics scenarios. (a) shows sea level change in meters from 2000 to the indicated times. (b) differences in predicted GMSL between simulations adopting 3-D viscoelastic and rigid Earth structures, expressed in meters and in percent difference (3-D - Rigid/3-D). (c) As in (b) but for differences between simulations adopting 3-D viscoelastic and elastic Earth structures. Note that these GMSL values are computed in the nested ice-sheet model simulations, just for the region of West Antarctica indicated by the pink line in Fig. 1a.

Movie S1. Animation showing the evolution of ice surface and bedrock elevations and grounding line position along a cross section through Thwaites glacier under RCP2.6 emissions scenario with MICI processes included.

Movie S2. Animation showing the evolution of ice surface and bedrock elevations and grounding line position along a cross section through Thwaites glacier under RCP8.5 emissions scenario with MICI processes included.

Movie S3. Animation showing the evolution of ice surface and bedrock elevations and grounding line position along a cross section through Thwaites glacier under RCP2.6 emissions scenario without MICI processes (labelled MISI).

Movie S4. Animation showing the evolution of ice surface and bedrock elevations and grounding line position along a cross section through Thwaites glacier under RCP8.5 emissions scenario without MICI processes (labelled MISI).

Movie S5. Animation showing the evolution of ice surface and bedrock elevations and grounding line position along a cross section through Pine Island glacier under RCP2.6 emissions scenario with MICI processes included.

Movie S6. Animation showing the evolution of ice surface and bedrock elevations and grounding line position along a cross section through Pine Island glacier under RCP8.5 emissions scenario with MICI processes included.

Movie S7. Animation showing the evolution of ice surface and bedrock elevations and grounding line position along a cross section through Pine Island glacier under RCP2.6 emissions scenario without MICI processes (labelled MISI).

Movie S8. Animation showing the evolution of ice surface and bedrock elevations and grounding line position along a cross section through Pine Island glacier under RCP8.5 emissions scenario without MICI processes (labelled MISI).
